# Stepwise pathway for early evolutionary assembly of dissimilatory sulfite and sulfate reduction

**DOI:** 10.1038/s41396-023-01477-y

**Published:** 2023-07-19

**Authors:** Sinje Neukirchen, Inês A. C. Pereira, Filipa L. Sousa

**Affiliations:** 1 Genome Evolution and Ecology Group, Department of Functional and Evolutionary Ecology, University of Vienna, Djerassiplatz 1, 1030 Vienna, Austria; 2 Instituto de Tecnologia Química e Biológica António Xavier, Universidade Nova de Lisboa, Av. da República, 2780-157 Oeiras, Portugal

**Keywords:** Molecular evolution, Phylogenetics, Archaeal genomics, Bacterial genomics, Bacterial evolution

## Abstract

Microbial dissimilatory sulfur metabolism utilizing dissimilatory sulfite reductases (Dsr) influenced the biochemical sulfur cycle during Earth’s history and the Dsr pathway is thought to be an ancient metabolic process. Here we performed comparative genomics, phylogenetic, and synteny analyses of several Dsr proteins involved in or associated with the Dsr pathway across over 195,000 prokaryotic metagenomes. The results point to an archaeal origin of the minimal DsrABCMK(N) protein set, having as primordial function sulfite reduction. The acquisition of additional Dsr proteins (DsrJOPT) increased the Dsr pathway complexity. *Archaeoglobus* would originally possess the archaeal-type Dsr pathway and the archaeal DsrAB proteins were replaced with the bacterial reductive-type version, possibly at the same time as the acquisition of the QmoABC and DsrD proteins. Further inventions of two Qmo complex types, which are more spread than previously thought, allowed microorganisms to use sulfate as electron acceptor. The ability to use the Dsr pathway for sulfur oxidation evolved at least twice, with *Chlorobi* and *Proteobacteria* being extant descendants of these two independent adaptations.

## Introduction

Microbial dissimilation of sulfur compounds has been, and still is, influencing the global biochemical sulfur and carbon cycles on Earth [1–5]. Dissimilatory sulfite/sulfate reduction is thought be one of the oldest energy conserving strategies [6] dated to the early Archean (3.47 Gya) [7]. A key protein in microbial sulfite reduction is the dissimilatory sulfite reductase (DsrAB), which is generally used as functional marker of this process in environmental and genomic studies [8–10]. In dissimilatory sulfate/sulfite-reducing microorganisms (SRM), several Dsr proteins are involved in the conversion of sulfite to sulfide. DsrAB produces a DsrC-trisulfide from sulfite and the DsrC protein. The DsrC-trisulfide is then reduced by the DsrMK(JOP) membrane complex recycling DsrC and releasing sulfide while coupling this reduction to energy conservation [11]. In some microorganisms, only a DsrMK complex is present [12, 13]. The *dsr* genes can also be found in dissimilatory sulfur-oxidizing bacteria (SOB), involved in the cytoplasmic oxidation of sulfane sulfur to sulfite by the reverse action of the DsrABCMKJOP proteins [14], where other proteins such as DsrEFH [15] and DsrL [16] are also involved. Here, DsrABCMK are defined as the minimal set of proteins necessary for the dissimilatory processing of sulfite in reductive, oxidative, and disproportionating metabolisms.

Several microorganisms are capable of dissimilatory sulfate reduction via sulfite to sulfide. The reduction of sulfate to sulfite thermodynamically requires the activation of sulfate with ATP to form APS (adenosine 5′-phosphosulfate) by the sulfate adenyl transferase Sat [17]. The APS reductase AprAB receives electrons from the quinone-interacting membrane complex QmoABC [18] and APS is reduced to sulfite serving as substrate of the Dsr cascade. In some SOB, the Sat-AprAB-Qmo cascade catalyzes the reverse reaction, although the interaction partner of AprAB can be replaced by the membrane protein AprM [19]. The bacterial reductive-type Dsr proteins are also present in microorganisms performing disproportionation of sulfur compounds for which the operative direction of the Dsr pathway is not clear, being proposed to be oxidative [20] and also reductive [21].

Previous analyses of DsrAB and AprAB phylogenies raised different evolutionary scenarios. Based on the limited availability of sulfate on early Earth, it was proposed that DsrAB was initially used to catalyze the oxidation of sulfide in SOB [22]. Further, AprAB proteins from SOB were thought to be the ancestral form, with SRM arising only after the accumulation of sulfate produced by SOB and/or after the oxygenation of the atmosphere [22]. In contrast, phylogenetic analyses of siroheme-containing sulfite reductases indicated a primordial function of DsrAB as sulfite reductase, with an ancestral siroheme-dependent sulfite reductase, lacking the fused ferredoxin-domain, existing before the duplication of DsrA and DsrB proteins [9, 23, 24]. Comparisons of 16S rRNA and DsrAB phylogenies of SRM indicated that *dsrAB* genes were mainly vertically inherited and to lesser extend laterally acquired [8, 9]. In line with the analysis of sulfite reductases with an initial reductive function, *dsrAB* genes were proposed to probably be either present before the split into the archaeal and bacterial domains or shortly after, invoking an early inter-domain lateral gene transfer (LGT) event [9, 24]. At that time, the known and sequenced diversity of archaeal SRM was limited and the presence of *dsr* genes in *Archaeoglobus* and *Aigarchaeota* was concluded to be the result of LGT events from SRM bacterial donors [9, 25], while the other archaeal DsrAB sequences represented a deeply branching archaeal type [9, 24]. With the discovery of this pathway in more lineages, newer studies of DsrAB phylogenies propose instead that the evolution via LGT is masking the origin of reductive *dsrAB* genes [8] and the lineage at the origin of this pathway may never be identified. In a recent study [26], based on the discovery of *Diaforarchaea* lineages containing the DsrABCMK apparatus and whose enrichment culture is sustained by HSO_3_^−^ but not SO_4_^2−^, the ancient existence of a sulfite-reducing (but not sulfate) Dsr system is proposed. Based on DsrAB and AprAB phylogenies, this ancient Dsr version was either present in the ancestor of the *Diaforarchaea* and *Thermoproteales* archaeal groups, prior to their diversification, or laterally acquired by the *Diaforarchaea* ancestor, followed by an intradomain transfer to *Thermoproteales*, from an unknown donor harboring a primitive Dsr system, whose nature is not addressed in the paper [26]. The existence of several bacterial lineages containing basal DsrAB sequences was attributed to recent LGTs [26].

Here we conducted large-scale phylogenetic analyses of more Dsr proteins involved in or associated with the dissimilatory metabolism of sulfite performed by SRM, sulfur disproportionating microorganisms (SDM), and SOB, including DsrAB, DsrMKJOP, DsrC, DsrEFH, DsrL, and DsrN involved in the amidation of the siroheme cofactor present in DsrAB proteins. Phylogenetics was coupled to comparative genomics and genomic neighborhood analysis in organisms possessing the minimal set of DsrABCMK proteins to elucidate the evolutionary history of the Dsr pathway and to provide a broader insight into the evolution of dissimilatory sulfur metabolism.

## Results

### DsrAB early branching lineages

It is generally accepted that the topology of *dsrAB* genes follows three major clades, namely, the basal branching archaeal reductive-type (including the second copy of *Moorella* spp.), the bacterial reductive-type including *Archaeoglobus*, and finally, the bacterial oxidative-type DsrAB proteins [9, 25, 27]. The second copy of *Moorella* spp. DsrAB sequences is used as root in many analyses. *Moorella* spp. are one of the few cultivated organisms in which two *dsrAB* copies are found. Specifically, in *M. thermoacetica*, we can find two clusters of *dsr* genes in different genomic regions and with different phylogenetic histories. One contains the *dsrAB* genes flanked by *dsrD*, and by the genes that constitute the DsrMKJOP complex and DsrT. In another region there is a second copy of the early-branching *dsrAB* genes encoded in the vicinity of *dsrC* and *dsrN* genes (Supplementary Table 1 and Supplementary Fig. 1). *M. thermoacetica* has been reported to utilize thiosulfate or DMSO as electron acceptors [28]. Further, both DsrAB copies were detected in a proteomic analysis confirming their expression in vivo [29] and suggesting their involvement in *Moorella* metabolism. However, to our knowledge, its Dsr system has not been investigated. If the Dsr system is operational, it is expected that, regardless of which DsrAB copy is active, DsrC and likely DsrN would be also participating in sulfite reduction.

**Fig. 1:**
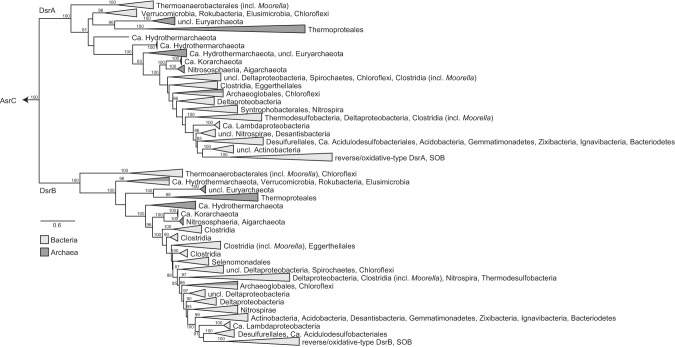
Phylogenetic reconstruction sulfite reductases. Paralogous rooting of DsrA and DsrB proteins using AsrC proteins as outgroup. Only ultrafast bootstrap values ≥80 are shown (model: LG + I + G4).

We have performed several phylogenetic reconstructions of DsrA(B) proteins using only sequences from complete genomes, the full metagenomic diversity of DsrA(B), and the paralogous rooting approach with the anaerobic sulfite reductase AsrC as outgroup for DsrA and DsrB. The inclusion of metagenomic data led to a change in topology, possibly reflecting the effect of sequence heterogeneity (both diversity and assembly/sequencing artefacts) on the alignment (see Supplementary Discussion). As expected, the paralogous rooting analysis retrieved the previously reported DsrA/DsrB phylogeny topology (Fig. 1 and Supplementary Fig. 2) with *Moorella* second copy branching early, followed by the archaeal reductive type and DsrAB proteins from unclassified taxa from different bacterial phyla such as *Candidatus* Rokubacteria, *Verrucomicrobia*, and *Elusimicrobia*. The next and highly supported clade contains proteins from the so-called bacterial reductive type, in which several archaeal sequences (*Ca*. Hydrothermarchaeota, *Ca*. Korarchaeota, *Thaumarchaeota*, *Aigarchaeota*) can be found branching at basal levels. The *Archaeoglobus* group within bacterial reductive-type DsrAB proteins were probably laterally acquired, as previously proposed [9, 30]. The last, highly supported split in both DsrA and DsrB clades contains bacterial oxidative-type proteins.

### Phylogeny of the cofactor modification DsrN protein

**Fig. 2:**
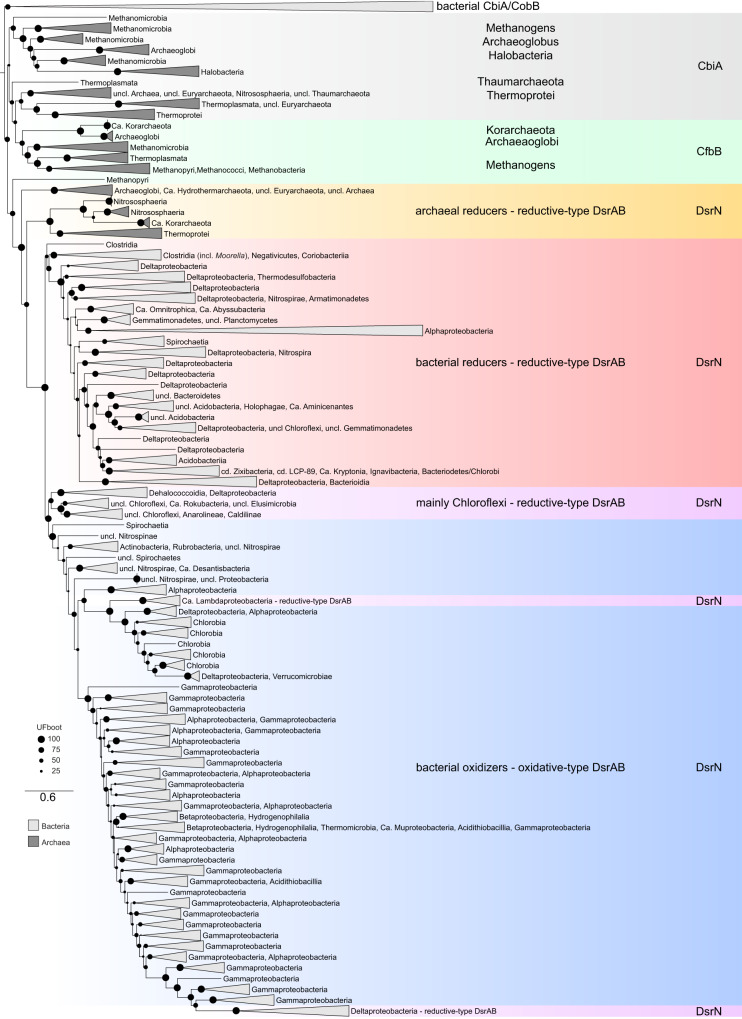
Phylogenetic reconstruction of DsrN proteins and homologous tetrapyrrole amidases. The different amidases are indicated with the color code. CbiA/CobB proteins, involved in cobalamin biosynthesis are highlighted in gray and the archaeal F_430_ cofactor biosynthesis protein CfbB in green. The three major types of DsrN proteins are highlighted in orange for archaeal reductive type proteins, red for bacterial reductive type proteins, and blue for oxidative type proteins. DsrN clades within the oxidative type containing assemblies with the reductive-type DsrAB proteins are indicated in pink. Ultrafast bootstrap values (UFboot) are represented with the black circles. Clades are colored in dark gray for archaea and in light gray for bacteria (model LG + I + G4).

In the SOB *Allochromatium vinosum*, it was shown that the prosthetic group of DsrAB proteins is siroheme amide, the amidated form of the siroheme cofactor of dissimilatory sulfite reductases [31]. It was concluded that the DsrN protein, a homolog of CbiA/CobB proteins involved in the amidation of cobyrinic acid [32], was necessary for the amidation of the siroheme cofactor and therefore important for dissimilatory sulfur metabolism in both SRM and SOB [31, 33]. CfbB, yet another homolog of CbiA, CobB, and DsrN proteins, performs the amidation of the F_430_ cofactor of the methyl-coenzyme M reductase involved in (reverse) methanogenesis and anaerobic alkane oxidation [34, 35]. In the phylogeny of the amidases of the different tetrapyrroles, the bacterial CbiA and CobB proteins were rooted by the minimal ancestor deviation (MAD) method [36] as outgroup (Fig. 2). The first clade at the other side of the root comprises archaeal CbiA proteins with the methanogenic CfbB proteins branching from within. The next clade consists of the DsrN proteins, which are also divided into three major types with archaeal DsrN proteins branching basal to the highly supported clade of DsrN proteins from bacterial SRM and SOB. In contrast to what is observed in the DsrAB phylogenies (Fig. 1), no bacterial sequences are basal to the archaeal DsrN clade, with *Moorella* spp. sequences branching deep within the bacterial clade, even though the *dsrN* gene is encoded in close vicinity to the *dsrAB* second copy in *M. thermoacetica* (Supplementary Fig. 1). This topology may indicate that DsrN proteins evolved within Archaea, from a duplication of an archaeal CfbB/CbiA protein, followed by functional adaptation. Within the bacterial DsrN clades, sequences from lineages such as *Chloroflexi* and *Ca*. Rokubacteria possessing a chimeric Dsr system, with both reductive-type and oxidative-type proteins, branch as a sister clade to the SOB’s DsrN proteins. The DsrN proteins from *Desulfurellales* and *Ca*. Acidulodesulfobacterales, which contain reductive-type DsrAB proteins, branch from within gammaproteobacterial SOB proteins. Some of the isolated *Desulfurellales* species were shown to use thiosulfate or elemental sulfur as electron acceptors and also to perform disproportionation of sulfur compounds [37, 38]. However, at least in *Desulfurella amilsii*, a comparative proteomic study has indicated that the DsrAB proteins are not involved in the disproportionation mechanism and instead seem to be involved in thiosulfate respiration [39]. DsrN proteins are highly conserved in organisms with the Dsr pathway and often encoded in close vicinity to other *dsr* genes, indicating its important role, being found in 1610 genomes with DsrABCMK proteins and absent only in 460 assemblies most of which (446) are incomplete.

### DsrMK phylogenies and genomic neighborhood

**Fig. 3:**
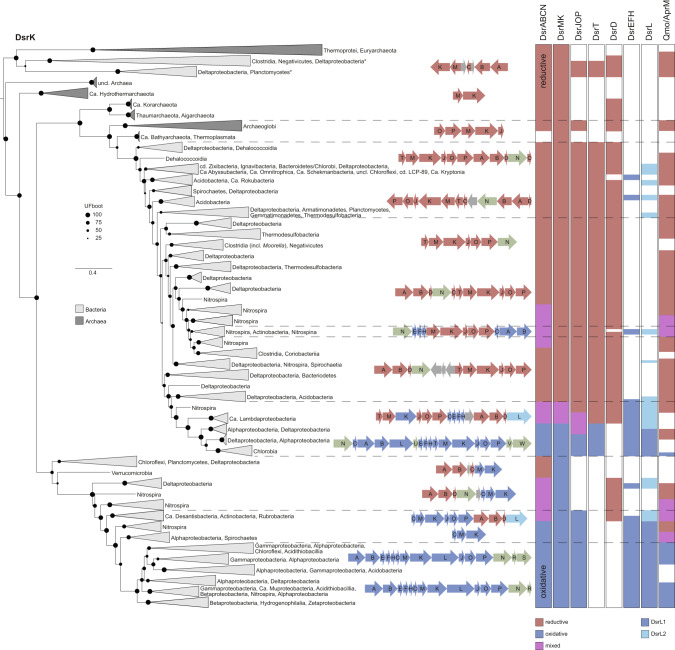
Phylogenetic reconstruction of DsrK and genomic organization of DsrK proteins in prokaryotes. Presence absence pattern of Dsr and Qmo/AprM proteins is shown on the right, colored in red for reductive-type, dark blue for oxidative-type proteins, and purple for mixed cases. DsrL is colored in dark and light blue corresponding to the DsrL1 and DsrL2 types [16]. Within the genomic arrangements, proteins without DiSCo type classification identified by similarity searches (DsrV,W,U,R,S and DsrN) are represented in green. Letters in genomic colocalizations indicate the subunit of Dsr proteins, colocalized non-Dsr proteins are shown in gray. *Organisms in which both DsrMK and DsrMKJOP exists, the genomic organization shown corresponds to the gene neighborhood of the sequence in the respective clade and the genomic content to the full set of genes present in the organism (model LG + I + G4).

The topology of the DsrM and DsrK phylogenies are mostly congruent (Fig. 3 and Supplementary Data). For clarity, only the DsrK phylogeny is shown. In DsrM proteins, two hemes are bound in a conserved transmembrane helix bundle [40–42]. Although the histidine residues remain highly conserved across DsrM sequences, the remaining segments are very divergent and only maintained the structural feature of the transmembrane helices. The cytosolic DsrK protein includes a CCG domain and two [4Fe-4S] cluster binding sites [13, 40, 42]. These features foster a higher sequence conservation leading to a better phylogenetic resolution. Nevertheless, the same three major clades are present in both DsrM and DsrK phylogenies: a basal archaeal reductive-type clade, a clade composed of mainly bacterial reductive-type proteins, and a bacterial oxidative-type clade. The topology not only follows a similar trend as DsrAB proteins with the three types, but is also tightly linked to the genomic arrangement of *dsrMK(JOP)* genes. The most basal clade consists of archaeal SRM and bacterial sequences from *Deltaproteobacteria* and *Clostridia* (not *Moorella* spp.) corresponding to a genomic organization with only *dsrMK* genes and no *dsrJOP* (Fig. 3 and Supplementary Fig. 1). These deltaproteobacterial lineages have additionally an DsrMKJOP version branching later in the phylogeny. The 74 bacterial assemblies clustering in the basal clade, either do not have any DsrAB protein identified (7 cases), or, in alternative (67), are branching within the bacterial reductive-type DsrA/B clades. On the contrary, the basal-branching and root-supported archaeal (*Thermoprotei*, *Euryarchaeota*) DsrK proteins have their corresponding DsrAB proteins either basal or basal to the bacterial reductive-type clade (Supplementary Figs. 1, 3–6). The remaining archaeal DsrK proteins are basal to the bacterial reductive-type clade and have DsrN encoded in the vicinity of the minimal DsrABCMK protein set. Most of these archaea are reported to be dissimilatory sulfite reducers or their genomic content comprises only the *dsrABCMKN* genes, lacking the Qmo proteins necessary for sulfate reduction (Supplementary Table 1) [26, 43, 44]. The next clade is the bacterial reductive type, in which, unexpectedly, *Archaeoglobi* lineages branch basal. From this split on, the full DsrMKJOP complex is present in a bacterial reductive-type Dsr gene cluster including *dsrD* and *dsrT*. In several bacterial lineages, the DsrABDNCTMKJOP proteins are encoded in one consecutive gene cluster with *dsrT* adjacent to *dsrM*.

The oxidative-type proteins from the well-known sulfur-oxidizing *Chlorobi* [45] branch within the bacterial-reductive type DsrK clade, together with uncharacterized proteobacterial lineages (e.g., *Ca*. Lambdaproteobacteria) which possess a chimeric *dsr* gene set including *dsrEFH* and *dsrL*. *Chlorobi* and *Ca*. Lambdaproteobacteria group together also in the DsrN, DsrEFH, DsrMJOP, and DsrC phylogenies (Fig. 2, Supplementary Figs. 7–9 and Supplementary Data). The *dsrMKJOP* genes in *Chlorobi* lineages are thought to have been laterally acquired from a sulfate/sulfite-reducing bacterial donor [14], and adapted to perform the oxidative instead of the reductive function.

The bacterial oxidative-type DsrK clade consists of a genomic neighborhood pattern similar to reductive-type clades. Several lineages, including *Desulfurellales* and *Ca*. Acidulodesulfobacterales, with reductive-type DsrAB, branch basal and possess only the oxidative-type *dsrMK* genes. The next clade is composed of sequences from lineages where the genes of the full oxidative-type DsrMKJOP complex are found in synteny with reductive-type *dsrAB* and *dsrD* genes. The last clade of DsrK proteins consists of typical proteobacterial SOB. As previously observed [46], the *dsr* genes necessary for dissimilatory oxidation of sulfane sulfur are consecutively encoded in one gene cluster (Fig. 3 and Supplementary Table 1).

### Phylogeny of DsrC and other Dsr proteins

In the DsrC phylogeny, the root separates reductive-type from oxidative-type DsrC proteins (Supplementary Fig. 7) with sequences from *Archaeoglobales* and *Ca*. Hydrothermarchaeota branching close to the root on the reductive-type side. The remaining reductive-type DsrC sequences are divided into two main clades. On one side, several archaeal lineages are basal to various bacterial subclades covering a taxonomic diversity ranging from non-monophyletic *Clostridia* to uncultured lineages such as *Ca*. Rokubacteria or *Spirochaetes*. The other side is dominated by *Thermodesulfobacteria* and *Deltaproteobacteria* including the *Desulfovibrio vulgaris* Hildenborough DsrC sequence. Recently, it was experimentally shown that, besides being involved in Dsr metabolism, *D. vulgaris* Hildenborough DsrC also interacts with the FlxABCD-HdrABC complex during fermentative growth [47]. The involvement in other metabolic processes might lead to functional changes that are reflected in the phylogeny by this clade separation. However, further experimental characterization is needed before such a generalization can be put forward, especially considering that DsrC proteins belonging to organisms containing the FlxABCD-HdrABC complex are also present in the other bacterial reductive-type DsrC clade. Sequences from *Desulfurellales* and *Ca*. Acidulodesulfobacterales branch close to the root and basal to the oxidative-type DsrC proteins. Some *Ca*. Acidulodesulfobacterales assemblies contain both reductive- and oxidative-type Dsr proteins and *Ca*. Acidulodesulfobacterales are possibly capable to perform both sulfate reduction and sulfide oxidation depending on oxygen levels [48]. Moreover, it can be seen that several *Nitrospirae* sequences branch with low phylogenetic resolution between *Desulfurellales*/*Ca*. Acidulodesulfobacterales and SOB sequences. Oxidative-type DsrC proteins from proteobacterial SOB are organized mostly in monophyletic clades with *Chlorobi*/*Ca*. Lambdaproteobacteria DsrC sequences as sister clade of gammaproteobacterial SOB.

The phylogenies of DsrJOP proteins are largely congruent and show the same general trend in which reductive-type proteins are separated from oxidative-type proteins by the estimated MAD root. In here, DsrO was selected as a representative of DsrJOP proteins due to its better phylogenetic resolution (Supplementary Data). DsrP is a cofactor-less membrane protein and maintains mainly structural conservation [40, 42, 49]. DsrP belongs to the PsrC/NrfD protein family whose phylogeny was shown to follow the number of transmembrane helices [49]. The periplasmic *c*-type DsrJ cytochromes [50] from SOB, SDM, and SRM have low sequence conservation which affects the quality of alignments. Hence, these phylogenetic reconstructions need to be interpreted with caution. In contrast, the DsrO protein binds several [4Fe-4S] clusters [40, 42] maintaining higher sequence conservation. DsrO together with DsrP forms a NrfCD-like redox module, also found in many members of the CISM family [51], which is proposed to function in a redox loop in anaerobic respiration [52]. The DsrO phylogeny was rooted by MAD separating the oxidative- from the reductive-type proteins, where two clades containing bacterial homologs are present, one of them, at a basal position. The reductive-type DsrO proteins are divided into several clades. DsrO proteins of archaea possessing the full DsrMKJOP complex (*Archaeoglobales* and *Ca*. Hydrothermarchaeota) branch in three clades, located between the root and the bacterial reductive-type proteins (Supplementary Fig. 8) and being intercalated by the two clades of homologous sequences. These homologous sequences are a group of proteins, whose genomic neighborhood varies between genes encoding for NrfD-domain-containing proteins or pseudo genes, thus their function is not clear. However, given the almost ubiquity of CISM proteins across prokaryotic lineages, when compared to the DsrOP distribution, it would be feasible to suggest that these modules would have been recruited from existing CISM families to enable novel catalytic functions [41].

DsrO proteins from various uncharacterized lineages (e.g., *Ca*. Rokubacteria, *Spirochaetes*, *Acidobacteria*, cd. Zixibacteria) branch between *Archaeoglobales* and the remaining bacterial reductive-type proteins. As in the DsrK phylogeny, the DsrO sequences from *Chlorobi* and *Ca*. Lambdaproteobacteria are within the bacterial reductive-type proteins, close to one of the *Deltaproteobacteria* and *Thermodesulfobacteria* clades. Sequences from *Nitrospirae*, *Clostridia*, *Thermodesulfobacteria*, and *Deltaproteobacteria* occur in multiple non-monophyletic clades, with *Morella* spp. sequences branching within the bacterial reductive-type clade. The oxidative-type DsrO proteins are dominated by two gammaproteobacterial clades, interspaced by a clade covering the remaining proteobacterial SOB. Sequences from *Ca*. Desantisbacteria, *Actinobacteria*, and *Nitrospirae* are basal to all oxidative-type proteins. Within these assemblies, both reductive- and oxidative-type proteins are found.

The phylogenies of DsrL and DsrE proteins, previously thought to be restricted to SOB, show a common pattern. The phylogeny of DsrL proteins is congruent with the one recently reported [16]. The root separates the so-called DsrL1 proteins from SOB and DsrL2 proteins from lineages with a chimeric Dsr system containing both oxidative-type and reductive-type proteins (including DsrD) (Supplementary Fig. 10). DsrL2 proteins are found in some lineages having also the DsrEFH complex. In the DsrE phylogeny (Supplementary Fig. 9), the sequences from DsrL2- and DsrL-1B-containing organisms form a distinct clade. The remaining clades of the DsrE phylogeny contain proteobacterial SOB possessing the oxidative-type Dsr pathway. The root position lies in a clade of DsrE-like proteins, mainly from *Gammaproteobacteria* representatives without Dsr proteins [53, 54].

### Intertwined evolution of QmoABC and AprAB

The QmoABC complex is the result of a functional reshuffling of modules found in methanogenic archaea (HdrA for QmoA and QmoB; and a dihemic heme module, found in several proteins including HdrE and HdrC in the case of QmoC [13, 41]). Recently, a large phylogenetic reconstruction of QmoAB and HdrA proteins showed the existence of at least two distinct evolutionary events leading to the assembly of QmoAB(C) complexes [43]. The expanded phylogenetic analysis to also QmoC, combined with sequence similarity analysis here reported, confirms the separation of QmoA, QmoB, and QmoC proteins into at least two types (Supplementary Figs. 11–13). Type I contains canonical QmoABC proteins from both SOB and SRM such as *Chlorobaculum tepidum*, *D. vulgaris*, or *A. fulgidus*. Type II *qmoAB* genes are more closely related to typical *hdrA* genes than to bacterial *qmoAB* genes and occur in the characterized sulfate reducers *V. moutnovskia* [43] and *Ammonifex degensii* [55]. Interestingly, both copies of QmoC proteins in *A. degensii* are of type II. We found that type II is also present in other organisms with or without Dsr and Apr proteins, so further experimental studies are required to clarify the functional role of these proteins. By comparing the phylogenetic reconstructions of AprAB and QmoABC (Supplementary Fig. 11), we observed that the consistent grouping of organisms is not just valid for the QmoAB-HdrBC case (see Supplementary Discussion), and in general, the groupings are found across the majority of the phylogenies. In addition, the co-evolution of AprAB and Qmo proteins can be traced by the consistent grouping of lineages in agreement with their syntenic arrangement (Supplementary Fig. 11).

## Discussion

We have performed a large-scale phylogenetic and comparative genomic analysis of the Dsr pathway and accessory proteins, and observed common trends in its evolution. These results, building on previous studies and also recent advances in the field, allows a broader insight regarding the evolution of dissimilatory sulfur metabolism.

### Assessment of early branching lineages across the phylogenies of the minimal set

Regarding the Dsr pathway, focusing on lineages that typically branch basal in DsrA/B phylogenetic reconstructions [8, 9, 26] leads to the conclusion that both Bacteria and Archaea branch early. Thus, the origin of this metabolic process could have been attributed to LUCA, or to either of the prokaryotic domains, with an early interdomain LGT event [9, 24]. With the discovery of previously unknown lineages harboring *dsr* genes through metagenomics, novel lineages appear branching early [8, 26] and changes in the relationships between the three types of DsrAB proteins (Fig. 1 and Supplementary Fig. 2) are observed. Besides DsrA/B, other proteins such as DsrC, DsrMK, or likely even DsrN are part of the minimal set found across all SRM, SDM, and SOB. Thus, their combined analysis can shed light on the evolution of this pathway and contribute to strengthening or rejecting current views on this topic.

Regarding archaeal reductive-type proteins, sequences from *Thermoproteales*, *Ca*. Hydrothermarchaeota, and several archaeal lineages (in where the *Diaforarchaea* proteins from Colman et al. [26] are included) are consistently branching basal or basal to the reductive-type bacterial clade within the DsrA/B, DsrN, and DsrM/K phylogenies (Figs. 1–3 and Supplementary Figs. 1, 3–6). In the DsrC phylogeny, archaeal sequences are either basal to the reductive-type DsrC proteins, or branch within this clade (see above, Supplementary Fig. 7). Thus, archaeal reductive-type proteins tend to be basal not only in the DsrAB phylogenies, but also across the reconstructions of the other proteins from the minimal set. However, and contrary to what is expected, the same pattern is not observed for the bacterial lineages found to be basal in DsrAB phylogenies (Supplementary Figs. 1, 3–6). For instance, while *Ca*. Rokubacteria and *Elusimicrobia* sequences are basal in DsrAB phylogenies, within the DsrMKC phylogenies, they group within the reductive-type bacterial clades, although in the *Elusimicrobia* assembly no DsrK was found. Within the *Chloroflexi* phylum, for one assembly (Caldilineae bacterium J123) DsrAB proteins were found within the basal archaeal reductive-type clades. However, the corresponding DsrC protein branches within the bacterial reductive-type clade, and the DsrMK and DsrN proteins occur in a as sister clade to the oxidative-type proteins. The basal *Chloroflexi* proteins in the DsrM phylogeny are from organisms in which no DsrAB proteins were identified. Proteins from *Desulfurellales* and *Ca*. Acidulodesulfobacterales group consistently in the same clades and are found close to the root only in the DsrC phylogeny (at the oxidative-type side). The basal branching of these lineages is not observed in DsrAB, DsrN, and DsrMK phylogenies (Supplementary Figs. 1, 3–6). Thus, the phylogenies of the minimal set show contradicting signals for the bacterial proteins, reflecting their different evolutionary histories with possibly mixed events of LGTs. For the metagenome-derived lineages, these inconsistencies can be explained by two different, but non-mutually exclusive, hypotheses: (1) a chimeric pathway assembly occurred, with the extant genomic content being the result of several LGT events, as also proposed in [8, 26]. In this case, and since the majority of the proteins are not found within basal clades, Occam’s razor would support a more recent acquisition of the *dsrAB* genes, excluding the origin of this pathway within these bacterial lineages; (2) the basal DsrAB proteins are the result of assembly artifacts (e.g., sequencing errors that would lead to basal positions due to long branch attraction). If so, only isolation, resequencing of the isolated microorganisms, and experimental characterization of the proteins could shed light on the in vivo function of the proteins and their primary structure. In the case of *Moorella* spp., with exception of the DsrAB second copy, the remaining *dsr* genes, including the flanking *dsrC* and *dsrN*, are not basal within the minimal set phylogenies (Supplementary Fig. 1).

The clear difference between archaeal and bacterial Dsr proteins in terms of consistent basal branching favors a scenario reflecting an early archaeal origin of a primordial archaeal reductive-type DsrABCMK(N) module (see Supplementary Information for discussion of alternative/previously proposed scenarios). In this case, the pathway was assembled in an early archaeal lineage, where it was kept in its most primitive form, and through interdomain LGT spread to Bacteria, where further LGT events occurred. Given the current data [26, 56] and the analysis presented here, we argue that this scenario seems to be the most parsimonious explanation. This is partially in agreement with recent reports by Colman et al. [26] which propose an archaeal origin of sulfite reduction within *Diaforarchaea*. The consistent placement of *Archaeoglobales* and other archaeal sequences in DsrC, DsrMK and DsrN phylogenies at the base of the reductive-type clades may indicate an even earlier origin of the sulfite-reducing minimal module in Archaea, at the ancestor of *Diaforarchaea* and *Archaeoglobus*, with an LGT event to *Thermoproteaceae*. Only later, *Archaeoglobus* acquired the sulfate reduction ability (see below). An alternative scenario would involve an origin of sulfite (but not sulfate) reduction in one of the ancestors of the taxa currently known to perform this metabolism (*Diaforarchaea*, *Thermoproteaceae*, or *Archaeoglobales*), followed by two intra-domain LGT within Archaea.

The ability to use sulfite or sulfate in several archaeal lineages was experimentally reevaluated by growth experiments with isolated microorganisms [43] previously reported to grow on sulfate [57–59] combined with proteomics analysis of *V. moutnovskia* binary culture in different growth conditions [43]. Based on phylogenetic analysis, the ability to utilize sulfate in *V. moutnovskia* was attributed to a later LGT event from bacteria, leading to the acquisition of the QmoABC complex and thus the re-invention of sulfate reduction in these lineages [43]. In addition, an enrichment culture containing *Diaforarchaea* lineages, containing an early archaeal type Dsr system, was tested and only produced sulfide when sulfite was provided [26]. This indicates that the existence of archaeal lineages with the ability to use sulfite [26, 43, 57–59], but not sulfate, at basal positions within the DsrABCMK and DsrN phylogenies (and DsrJOP), may represent a relic of the ancestral version of the pathway. These findings, along with geochemical records where sulfite is propose to have been more readily accessible than sulfate [60], point to sulfite as the likely initial substrate for archaeal Dsr-containing organisms [26, 43], as also observed in our data.

### Modular increase of Dsr pathway complexity

The large-scale analysis of Dsr proteins, along with reported scenarios on their evolution, has led to potential connections with other metabolic processes such as methanogenesis through shared homologous modules. Here, the transition from sulfite to sulfate reduction and sulfane sulfur oxidation is discussed. Siroheme-containing sulfite reductases are involved in assimilatory and dissimilatory processes and, together with assimilatory nitrite reductases and the Fsr and Dsr-LP proteins from methanogens, part of the siroheme-containing reductases family [61–63]. The simpler version of this family (in some cases including dissimilatory DsrAB proteins) was proposed to have been present before the bacterial and archaeal divide [9, 23, 24, 64]. However, due to increased diversity and functional characterization of other members [65–67], the substrate of the primordial module is unknown. Many cultivated methanogens are able to assimilate sulfide as sole sulfur source [68]. This was perhaps the initial way early microbes incorporated sulfur into biomass. For energetic reasons, spending ATP for assimilatory purposes in environments, where sulfide would be present, would not be the best strategy. Thus, a sulfite detoxification role for the primordial DsrAB/sulfite reductase module may make more sense.

**Fig. 4:**
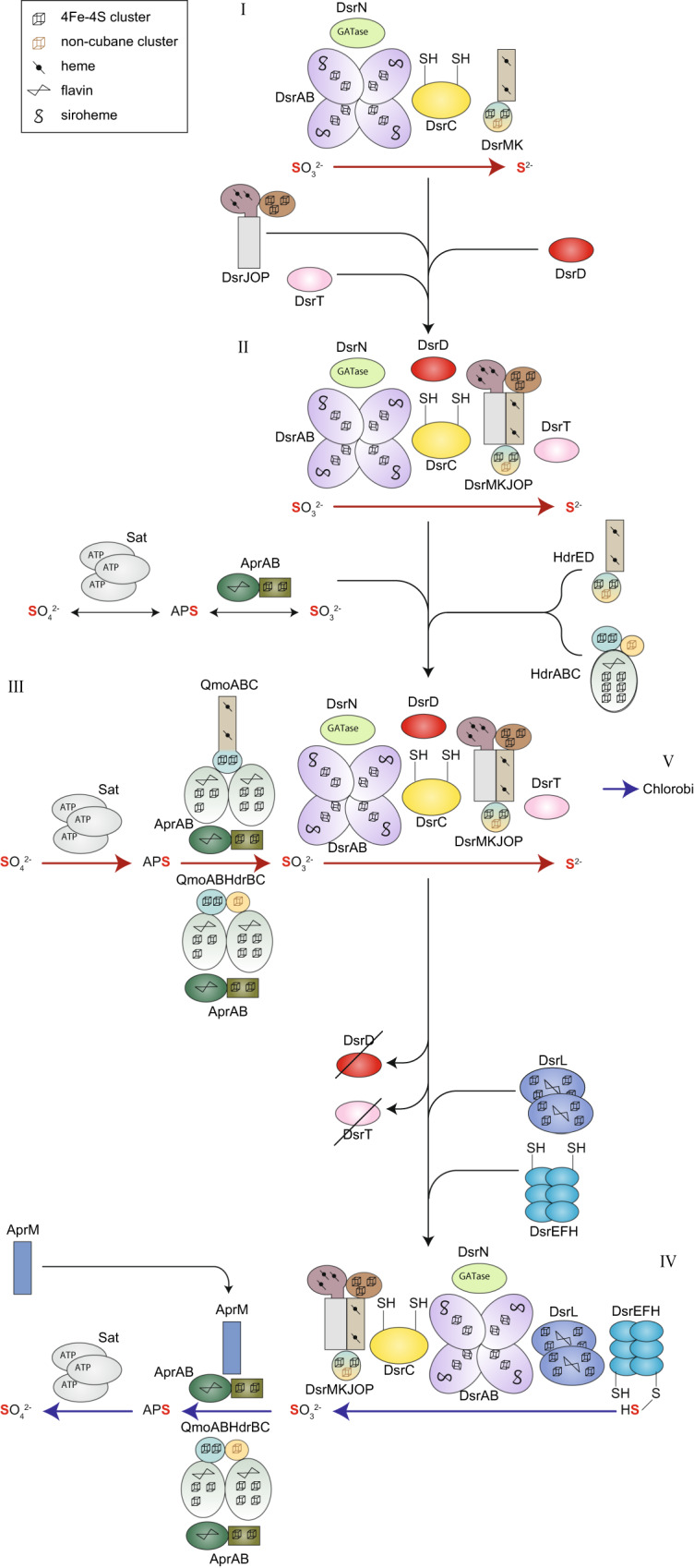
Modular nature and increase in complexity of the Dsr cascade over time. Schematic representation of the proposed evolutionary scenario. Homologous proteins are indicated with the same color code. Red arrows—reduction, blue arrows—oxidation. ATP ATP-binding site, GATase glutamine amido transferase domain. Other proteins such rhodanese and TusA, likely involved in S transfer to DsrEFH, are not drawn for simplicity.

The coupling of the DsrC protein to the DsrMK complex allowed for energy conservation through reduction of the DsrC-trisulfide by the DsrMK membrane complex and an enhanced sulfite reduction activity [11, 21]. This simple version of the Dsr system is still present in extant archaea utilizing DsrABCMK for sulfite reduction to sulfide [26, 43] (Fig. 4 Step I and Supplementary Table 1). Additionally, archaeal DsrN proteins are basal to the bacterial DsrN clades, indicating that the biosynthesis of siroheme amides most likely co-evolved with the catalyst DsrAB. Most SRM, besides siroheme, also have the heme biosynthesis via the siroheme pathway, which is the main heme biosynthetic route present in Archaea [69, 70]. The abiotic or biotic existence of hemes is a prerequisite for the functional assembly of the minimal module since in DsrM two heme cofactors are present [40, 42]. Although speculative, this may further support an origin of the minimal set within Archaea, with an early inter-domain transfer of not only Dsr proteins but possibly also of the heme biosynthesis via siroheme to bacterial SRM. However, further analyses are necessary to test this hypothesis. Of note, within sulfur oxidizers, other heme biosynthesis pathways exist.

Over time, the minimal set was extended and DsrJOP proteins were recruited and together transferred to the bacterial domain. With the addition of DsrD, activation of sulfite reduction by DsrAB [21] became possible. The complete DsrMKJOP complex possibly enabled periplasmic electron transfer or more efficient energy conservation [40, 42] (Fig. 4 Step II).

This protein set exists in the more evolved sulfate-reducing *Archaeoglobus* and in *Ca*. Hydrothermarchaeota (Supplementary Table 1). The *Archaeoglobus* Dsr pathway was thought to have been laterally acquired from bacterial reducers [9, 30]. However, only the DsrAB proteins show this evolutionary signature (Fig. 1 and Supplementary Figs. 1–6), while the DsrCMKJOP and DsrN proteins represent a more ancestral version. Our data point to an acquisition (replacement) of *Archaeoglobus* DsrAB proteins from a bacterial SRM, in an *Archaeoglobales* ancestor which, we suggest, already contained the archaeal-type DsrABCMKJOPN protein set. Although not clear what would have been the evolutionary pressure to exchange the archaeal for the bacterial DsrAB version, this could have been the result of a simple synonymous replacement, as observed for other proteins [10, 71]. In addition, *Archaeoglobus*, contrary to the majority of Dsr-containing archaea, also has the DsrD protein known to be present in bacterial SRM. The simultaneous acquisition of *dsrD* and bacterial *dsrAB* genes, allowing for more efficient sulfite reduction, could have been the selective pressure to keep the bacterial DsrAB version adapted to interact with DsrD within *Archaeoglobus*. A recent study, using crystallographic and modeled DsrAB structures [56], proposed similar allosteric pathways at the heterodimeric DsrAB interface. The more evolved bacterial-type proteins, including *A. fulgidus*, could have allowed coupling to allosteric activation by DsrD [21, 56].

In bacterial reducers, the DsrABCD-DsrMKJOP proteins are co-distributed with DsrT, which, based on homology [8], may regulate *dsrMKJOP* expression, constituting a more advanced Dsr system already with possibly gene regulation and allosteric activation of DsrAB by DsrD [21, 56]. The Dsr pathway in bacterial reducers was initially believed to have a complex evolutionary history with *Deltaproteobacteria* having the highest number of laterally acquired *dsr* genes [8]. However, the recent reclassification of *Deltaproteobacteria* as *Desulfobacterota* [72] revealed that sulfite reduction in bacteria was mostly vertically inherited with the main inconsistencies present in *Clostridia* which is per se a taxonomically polyphyletic group [73].

Based on previous studies and the results presented here, an updated evolutionary path for dissimilatory sulfate reduction, considering the function of AprAB and Qmo proteins is presented. The separation observed in AprAB phylogenies and the presence of full-length, canonical AprAB and Sat proteins in sulfite-reducing archaea incapable of sulfate reduction and in organisms devoid of Dsr proteins suggests their initial role may have been in sulfate assimilation [43, 53] (Supplementary Fig. 11). In addition, since sulfite can also be inhibitory or toxic to microorganisms, the AprAB innovation may have allowed its detoxification with oxidation of sulfite to APS, as previously proposed [74], although usually sulfite is detoxified through reduction. While Sat proteins are used by some extant organisms in assimilatory functions, expending ATP for assimilatory purposes using the Sat-AprAB cascade would not be energetic efficient, especially considering the likely higher abundance of sulfide over sulfate on early Earth [60]. However, the ancestral reductive function in sulfate assimilation cannot be ruled out as the archaeal/*Thermoprotei* AprA sequences branch close to the root [43] (Supplementary Fig. 11). In addition, in *Ferroglobus placidus* the *sat* and *aprA-like* genes may be part of an assimilatory sulfur mechanism [53] during iron respiration [75]. It is neither clear when the coupling of the Sat and AprAB proteins occurred nor their initial function (dissimilatory or assimilatory). Since the phylogenetic signal of these proteins is unclear, the primordial proteins might have had a broader and unspecific catalytic activity. Moreover, AprA is a flavoprotein that belongs to the so-called fumarate reductase/succinate dehydrogenase superfamily [76, 77]. This widespread module has been reused in different enzyme architectures and the initial substrate of the ancestral module is also unknown.

Qmo proteins transfer electrons to the AprAB complex necessary for APS catalysis [18] and the Qmo complex is present within sulfate reducers in different arrangements. The general configuration QmoABC resembles a reshuffling of the HdrABC and HdrED heterodisulfide reductase complexes [13], the latter, present in more evolved heme-bearing methanogens [78]. The QmoABC complex is believed to couple APS reduction with energy conservation [12, 18]. The alternative QmoAB-HdrBC or QmoAB-HdrD complexes are likely present in Gram-positive sulfate reducers [79] and, as reported here, also in some *Deltaproteobacteria*, *Clostridia*, and several unclassified lineages (Supplementary Fig. 11). Due to the absence of QmoC, it is thought that the coupling to membrane electron transfer is not possible in those organisms. Although the time of the Sat-AprAB invention remains to be elucidated, the merge of the Sat-AprAB-Qmo proteins with the Dsr pathway occurred probably after the evolution of the Dsr pathway to a more advanced DsrMKJOPT system (Fig. 4 Step III and Supplementary Discussion).

Over time, the recruitment of additional Dsr proteins such as DsrEFH and DsrL, and the functional evolution of the remaining Dsr proteins to catalyze the reverse reactions, allowed for reversal of the pathway enabling sulfur species oxidation in SOB, while the SRM-specific proteins DsrD and DsrT were lost (Fig. 4 Step IV). This later adaptation could have been the result of environmental changes (global or local) such as increasing levels of environmental redox potentials or pH [1, 80]. Comparison of *dsrAB* genes and 16S rRNA phylogenies also indicates a vertical inheritance of the Dsr pathway in SOB [9]. Sulfur oxidation to sulfate in lineages such as *Chlorobi* has a different evolutionary history than the pathway in proteobacterial SOB such as *A. vinosum*. *Chlorobi* species possess the QmoABC complex, including the QmoC subunit that is usually only present in SRM, while other SOB possess the QmoAB-HdrBC complex [19, 43, 53] or AprM [19]. Additionally, *Chlorobi* lineages encode also for the DsrT protein, and the DsrMKJOP and AprAB proteins are more similar to the ones from SRM [43] (Fig. 3, Supplementary Fig. 8 and Supplementary Data). *Chlorobi*-related lineages probably acquired the Sat-AprAB-QmoABC cascade and the DsrT-DsrMKJOP proteins (with/without DsrABCD) via an LGT from a sulfate reducer and the oxidative-type DsrABCN-DsrEFH-DsrL genes from an SOB. Environmental pressures made the ancestor of *Chlorobi* to adapt the remaining Dsr proteins for oxidative catalysis (Fig. 4 Step V). Overall, this indicates two independent paths in the evolution of the Dsr pathway toward sulfur oxidation present in the currently known diversity of SOB.

Microbial disproportionation of sulfur compounds such as elemental sulfur, sulfite, or thiosulfate using the Dsr pathway is a process that involves the simultaneous formation of sulfate and sulfide as end products, although the mechanism is not fully understood [81–83]. The SDM’s Dsr proteins from e.g., *Desulfurivibrio alkaliphilus* are phylogenetically indistinguishable from reductive-type bacterial Dsr proteins and do not form monophyletic clades. This means that with the current knowledge regarding SDM, genomic content in terms of Dsr proteins or their phylogenies, it is not possible to distinguish between SDM and SRM and to determine with certainty the order of appearance of these two metabolic processes. Nevertheless, the patchy taxonomic distribution of known SDM across the bacterial domain suggests separate mechanistic adaptations to perform sulfur disproportionation and favors several independent events involving additional proteins in the transport, regulation, and chemical transformations of sulfur compounds within the cell [84].

A possible mechanism for sulfur disproportionation may involve the Dsr pathway of SDM operating in the reductive direction (producing sulfide from sulfite) with the reversible Sat-AprAB-Qmo cascade oxidizing intracellular sulfite to sulfate as it has been proposed [21, 81, 82]. The bifurcation of sulfite in two different catalytic directions would be consistent with the sequence similarity of Dsr proteins from SDM and SRM, and would also be in agreement with the sulfate and sulfide production measured in sulfur disproportionation studies [20, 85, 86]. Further studies are necessary to clarify the in vivo operative function of the SDM enzymes, elucidate the nature of the sulfur intermediates in sulfur disproportionation, and the role of additional proteins such as Sqr, Psr/Phs, and rhodaneses [39, 84, 87]. Moreover, cultivation of early branching lineages with chimeric Dsr systems such as *Ca*. Rokubacteria and *Verrucomicrobia* could elucidate if these organisms are able to perform disproportionation and fully clarify their genomic content.

The reuse of the same building blocks to perform new functions is recurrent in biology as can be seen in the CISM or Hdr enzyme families [13, 41]. In addition, corrins (cobalamin and siroheme) and iron-sulfur centers are thought to be ancient cofactors [6] and by large-scale phylogenetic analysis proposed to have been present in the last universal common ancestor LUCA [88]. This supports an early archaeal invention of sulfite reduction followed by LGT to Bacteria, via recruitment of existing modules (some from methanogens), in a scenario in which sulfite reducers and Earth have been co-evolving for a long time.

## Conclusion

The combined investigation of the large-scale phylogenetic reconstructions of Dsr proteins, gene co-occurrence, and synteny analysis showed common trends in the evolution of the Dsr pathway. Our data supports the evolution of the sulfite reduction minimal module (DsrABCMK and DsrN) including DsrJOP within Archaea with an early lateral gene transfer event to Bacteria where the pathway evolved mainly vertically. The invention of sulfate reduction occurred by the recruitment of the Sat-AprAB and Qmo complexes, the latter from more evolved heme-containing methanogens. Extant sulfite/sulfate reducers share the same environments with methanogenic archaea, and it is plausible to assume that they have been sharing it for a long time. Sulfate reduction in Archaea evolved by (at least) two independent interdomain LGT events, one to *Archaeoglobus* ancestor (with replacement of DsrAB proteins and acquisition of DsrD and the QmoABC complex) and another to an ancestor of *Vulcanisaeta*. Our analysis further identified two independent evolutionary paths for the adaptation of a sulfate reducer into an SOB. For proteobacterial SOB, the evolution of the Dsr pathway seems to be also mostly vertical. Within lineages such as *Chlorobi* the Dsr pathway and the Sat-AprAB-QmoABC cascade were probably acquired from SRM while DsrABCEFHL were gained via LGT from SOB and together adapted for oxidative catalysis.

By taking the evolutionary history of each gene into consideration, a possible evolutionary path for the microbial ability to utilize sulfur compounds using the Dsr pathway is proposed, where inter- and intra-domain transfers as well as several functional adaptations and replacements are put forward.

## Methods

### Genomic dataset and identification of Dsr proteins using DiSCo

A dataset comprising 195,878 (meta)genomic assemblies (3131 archaea and 192,747 bacteria from where 356 archaeal and 15,594 bacterial assemblies correspond to complete genomes) was analyzed with DiSCo, as previously reported [53]. Briefly, all available prokaryotic genome assemblies with annotated protein sequences were retrieved from both NCBI RefSeq and GenBank databases in 2019. Additional assemblies were added based on recent literature, for details see [53]. This large dataset was used for similarity searches of additional proteins (see below). The quality of metagenomes was estimated with domain specific single copy markers following Rinke and colleagues [89]. The tool DiSCo was run against the genomic dataset and identified Dsr sequences were combined per Dsr protein. In total, 2070 genomes had at least one DiSCo hit to one protein of the minimal set DsrABCMK and were used for synteny analysis.

### Paralogous rooting

The 15,950 complete prokaryotic assemblies were screened for the presence of the anaerobic sulfite reductase AsrC. The TigrFam [90] (release 15) HMM profile TIGR02912 AsrC was run against each genome with the profile-specific gathering threshold using hmmsearch [91] (version 3.3). The identified AsrC protein sequences and the DsrA and DsrB sequences identified using DiSCo were used for a combined multiple sequence alignment and phylogenetic reconstructions using the paralogous sulfite reductase AsrC as an outgroup.

### Similarity search for additional Dsr proteins

Selected proteins such as DsrN and DsrMK were used as queries for a similarity search using diamond blastp [92] (version v2.0.5.143). To distinguish from homologous protein complexes with similar domain architecture, related protein complexes such as NarGHI, HdrED, multi-cytochrome membrane complexes Hmc, Tmc, and Ohc, as well as homologs of cobyrinic acid *a*,*c*-diamide synthase (CbiA, CobB, CfbB) were added to the search (Supplementary Table 2). Diamond blastp was run with all target hits (option -k 0) in the ultra-sensitive mode with the selected queries against 15,950 complete genomes and against the 2070 genomes with hits to at least one protein of the minimal set DsrABCMK. Diamond blastp hits were filtered for the best hit per query sequence using a ≥25% local identity and a ≤10^−10^ *E*-value threshold. Identified sequences were used for synteny analysis in the 2070 genomes containing DsrABCMK proteins. Further, sequences identified by DsrN, CbiA, CobB, and CfbB query sequences were used for a combined phylogeny of amidases of the different tetrapyrroles.

### Phylogenetic reconstructions

Sequences within one protein set were all-vs.-all globally aligned with the Needleman-Wunsch algorithm implemented in needleall [93] (Emboss [94] package 6.6.0, default gap penalties) and pairwise global identities above 90% were used to cluster each protein set using MCL [95] (version 14.137, inflation rate 2.0). To reduce redundancy, only the longest sequence per genus per MCL cluster was kept and used for multiple sequence alignments. These sequences were additionally filtered and only sequences derived from complete genomic assemblies were kept to calculate a second multiple sequence alignment (Supplementary Table 3).

The multiple sequence alignments were calculated using Clustal Omega (version 1.2.3) [96] with both 100 HMM and 100 guide tree iterations (output order = tree order) and trimmed with trimal [97] (version v1.4.rev22) using a 95% gap-threshold (Supplementary Table 4). Phylogenies were reconstructed with iqtree [98] (version 1.6.12) using the best model selection [99] and with the model LG + I + G4 (Supplementary Table 5). The phylogenies were built with 1000 ultrafast bootstraps [100], the SH-like approximate likelihood test [101] with 1000 replicates, and an approximate Bayes test [102]. A maximum number of 5000 iterations and the nearest neighbor interchange were used to optimize ultrafast bootstrap phylogenies. All phylogenies were rooted with the minimal ancestor deviation method MAD [36].

### Synteny analysis of Dsr proteins

Genomes with hits to at least one protein of the minimal set DsrABCMK were used for synteny analysis. The 33,490 hits obtained by DiSCo and the 25,855 diamond blastp hits present in the 2070 genomes containing DsrABCMK were mapped to their gene location files and proteins encoded consecutively with a maximum distance of four genes between two genes coding for DiSCo/diamond hits were extracted and plotted using genoplotR [103].

### Comparative analysis of Apr and Qmo proteins

AprA/B and/or QmoA/B/C sequences were identified (using DiSCo) in 563 (meta)genomes lacking the Dsr pathway. Following the analysis of the 2070 genomes with hits to the minimal set DsrABCMK, the 563 Apr/Qmo-containing genomes were screened for the selected queries (Supplementary Table 2) using diamond blastp [92] keeping the execution parameters and filtering criteria (see above). The 1446 diamond blastp hits and 2940 DiSCo hits were used for synteny analysis. In total, 258 QmoA/HdrA-like sequences were found encoded in close proximity to *qmoB* genes. Thus, QmoA and QmoB DiSCo hits and the co-syntenic QmoA/HdrA-like proteins were all-vs.-all globally aligned with needleall [93] for similarity analysis. QmoC sequences were also all-vs.-all globally aligned and the identities for both QmoA/HdrA-like/QmoB and QmoC sequences sets were hierarchically clustered (euclidean distance, complete clustering method) and plotted in R using the pheatmap package (version 1.0.12, https://CRAN.R-project.org/package=pheatmap).

Maximum likelihood phylogenies were reconstructed for AprA, AprB and QmoA, QmoB, and QmoC protein sequences using iqtree [98]. The strategy was analogous to the Dsr phylogenies applying 90% global identity redundancy reduction, using sequences from only complete genomes, trimmed alignments, different model selection, calculating branch support values, and rooting by MAD (see above). For the AprA, QmoA, and QmoB phylogenies homologous sequences were included and used for outgroup rooting. In the case of AprA, 81 succinate dehydrogenase/fumarate reductase sequences covering the know diversity [77] were selected. In the case of QmoA and QmoB, 14 HdrA sequences, used to build the different HdrA models implemented in DiSCo [53], were used as outgroup.

## Supplementary information

Supplementary Information

Supplementary Table 1

## Data Availability

The sequences, alignments, and phylogenies at the basis of Figs. 1–3 and Supplementary Figs. 1–11 are available in Figshare doi.org/10.6084/m9.figshare.20766064

## Code availability

The small scripts used in this paper are available at doi.org/10.6084/m9.figshare.20766064
