# Supplementary material for: Stepwise pathway for early evolutionary assembly of dissimilatory sulfite and sulfate reduction
Source: ISME J. 2023 Jul 19;17(10):1680–92. doi: 10.1038/s41396-023-01477-y (PMC10504309; doi:10.1038/s41396-023-01477-y)
Supplement: Supplementary file 1 — Supplementary Information [file 41396_2023_1477_MOESM1_ESM.pdf]

Supplementary Information

Stepwise pathway for early evolutionary assembly of dissimilatory sulfite and sulfate reduction

Sinje Neukirchen[1], Inês A. C. Pereira[2], Filipa L. Sousa[1]*

[1] Genome Evolution and Ecology Group, Department of Functional and Evolutionary Ecology, University of Vienna, Djerassiplatz 1, 1030 Wien, Austria

[2] Instituto de Tecnologia Química e Biológica António Xavier, Universidade Nova de Lisboa, Av. da República, 2780-157 Oeiras, Portugal.

*corresponding author: filipa.sousa@univie.ac.at

Supplementary discussion

Tree reconstruction statistics

For each protein set four phylogenies were reconstructed with firstly running the best model selection [1] (Supplementary Table 5) and, in the second phylogeny, with the amino acid substitution model LG+I+G4. An additional set of phylogenies was performed, with a reduction of sequences to proteins encoded in only complete genomes, to investigate the impact of metagenome-derived sequences (Supplementary Table 3). Phylogenies with only sequences from complete genomes were also computed with the model LG+I+G4 and additionally with the ModelFinder of IQ-tree [1]. 24 out 42 phylogenies were reconstructed with the identically chosen best model LG+I+G4 according to BIC (Bayesian information criterion), in seven cases the model WAG+I+G4 was chosen. In the remaining 17 cases the best model was always a LG model [2] with different combinations of parameters including the proportion of invariable sites +I, empirical frequencies +F, and discrete Gamma model with four categories +G4 (Supplementary Table 5).

Four phylogenies were reconstructed for each of the 15 Dsr proteins, however, some phylogenetic reconstructions needed to be excluded from further analysis. The proteins DsrE, DsrF, and DsrH belong to the DsrEFH sulfurtransferase complex [3]. The DsrE proteins bind sulfur with an conserved cysteine residue and transfer it to the DsrC protein [3]. Although DsrH has also one conserved cysteine, DsrE proteins are directly involved in the transfer and maintain a stronger phylogenetic signal. In contrast, the small DsrF and DsrH protein sequences create ‘star-like’ topologies and do not allow conclusive results. The same issue exists for the small sequences of DsrT proteins. Another problem occurs for DsrD proteins. The around 80 amino acids long DsrD sequences (Supplementary Table 4) have only low all-vs.-all sequence similarities, mostly below 25% global identity. Thus, the phylogeny creates artifacts with highly supported clades corresponding to the sequence identities below 25%.

Effect of metagenomic data in DsrA(B) phylogenies

We used three different approaches to reconstruct the phylogenies of DsrA(B) proteins: using sequences only from complete genomes, including all metagenomic diversity and using the paralogous rooting analysis. Both DsrA and DsrB clades mirror each other with the main differences being in missing representatives in either of the clades since multiple metagenome-derived lineages encode for only one of the two proteins due to their genome incompleteness. Further, some clades within the bacterial reductive-type DsrA and DsrB proteins have low phylogenetic resolution and are poorly supported.

While two of our methodological approaches retrieved the expected phylogeny, the inclusion of the extended diversity of Dsr protein sequences within a larger phylogeny led to a changed topology. In this phylogeny, the archaeal reductive-type DsrA proteins branch from within the bacterial reductive-type DsrA proteins (Supplementary Fig. 2a), instead of being in-between the reductive-type and oxidative-type bacterial clades (Supplementary Fig. 2b), as observed previously [4–6]. Here, the MAD method [7] separates the proteins by function, with the root between oxidative-type and reductive-type proteins (Supplementary Fig. 2a). Although not discussed, a similar topology was also found in a recent study in which several new Dsr-containing lineages were identified[8].

Alternative scenarios regarding the origin of DsrABCMK(N)-dependent sulfite reduction in Archaea

Besides the assembly of the DsrABC, DsrMK and DsrN pathway in Archaea, leading to an archaeal origin of this pathway (but not necessarily of the ancestral modules at the origin of each one of these protein families), alternative hypotheses were or can be put forward regarding the appearance of the ancestral module: i) the ancestral version of the pathway was present within the last universal common ancestor. This version would have been maintained within the archaeal domain and lost in the majority of Bacteria, with the ancestral *dsrAB* genes being kept only in *Moorella* spp. (2nd copy) and the ancestral *dsrMK* in the remaining *Clostridia* representatives. This hypothesis is not supported by our as well as other's analyses [4, 6, 9].

Briefly, although sulfite and sulfate records possibly trace back to 3.47 Gya [10], it is though that more reduced compounds (in this case sulfide) would have been the predominant sulfur species at the time of the origin of Life [11]. Taking into account the early-branching lineage analysis of the minimal set, this hypothesis is further unlikely, as it invokes multiple losses and regains (replacements) of the same metabolic pathway within Bacteria, especially within *Clostridia* genera.

Another hypothesis would be for the pathway to have originated within a so far unidentified or extinct bacterial lineage, and to have been acquired early in Archaea by possibly several interdomain LGT events. In this case, within Bacteria, the pathway would have evolved faster that in Archaea, where multiple events of gene losses, LGT, and replacements would have occurred. This hypothesis is unlikely as only the genes from archaeal assemblies, and not Bacteria, consistently branch basal within the phylogenies presented here (Supplementary Fig. 1, Supplementary Figs. 3–6).

Intertwined evolution of QmoABC and AprAB

The AprAB-Sat-Qmo complexes are necessary for sulfate activation to APS and its reduction to sulfite [12–16]. The AprA(B) phylogenies show a basal clade containing archaeal-type AprA(B) from sulfite reducers (where the sulfate reducers *Vulcanisaeta moutnovskia* [14] is included) having as sister clade SOB with the AprM protein. On the other side are several AprA clades of QmoABC-containing lineages, with AprAB sequences from *Chlorobi* SOB (with the QmoABC complex) branching in between. Other AprA from SOB with the QmoAB-HdrBC complex group in a later diverged clade. This functional separation into clades corresponding to the presence (or absence) of the electron transfer units Qmo and AprM was also previously reported [14, 17] . Here, we have further observed that besides the AprA clades corresponding to the QmoABC complex, a clade containing putative sulfate reducers, with variations of the canonical QmoABC complex is also found (Supplementary Figure 11) where the QmoAB-HdrD/HdrBC from *Clostridia*, *Deltaproteobacteria*, and several unclassified lineages are found. This tends to be associated with the synteny of AprAB linked to Qmo clades, sometimes with

Sat in one genomic arrangement. For instance, AprAB clades of *Deltaproteobacteria* are divided into three major clades according to their taxonomic order, having *Thermodesulfobacteria* as sister clade. This organization occurs also in QmoB and QmoC phylogenies, and corresponds to the Sat-AprAB-[typeI-QmoABC] operon.

Based on our synteny analysis, in most bacteria where the type I QmoABC complex is present, the DsrJOP, DsrT, and DsrD proteins are co-distributed (Fig 3., Supplementary Table 1). An exception to this observed trend is found in *Ca*. Acidulodesulfobacterales and *Nitrospirae* that branch basal to the oxidative-type DsrK proteins (Fig. 3.). In these lineages, the DsrMKJOP complex and the Sat-AprAB-QmoABC cascade are incomplete, and, if present, are a mixture of both reductive- and oxidative-type proteins. *Ca*. Acidulodesulfobacterales were proposed to be able to switch between oxidation and reduction of sulfur compounds [18], which may also be the case for these *Nitrospirae* lineages. These correspond to exceptions of this grouping, where *Ca*. Acidulodesulfobacterales and *Nitrospira* present in AprAB do not form a consistent clade across Qmo phylogenies (represented in green in Supplementary Figure 11), since the individual subunits of their Qmo complex are from type-I (QmoAB) and type-II (QmoC) in the case of *Ca*. Acidulodesulfobacterales. In summary, the overall topologies of AprA, QmoB, and QmoC (Supplementary Figure 11) show the existence of six major groups in AprAB phylogenies, four of which are also found within QmoABC proteins indicating their co-evolution over time.

Supplementary Table 1 | Syntenic regions of DiSCo/diamond blastp hits across 2,070 genomes with at least one hit to the minimal protein set DsrABCMK. Each syntenic region is separated by a semicolon. Proteins with hits for two non-homologous proteins were identified as fusion proteins and are indicated with ProteinX+ProteinY. Hits to AsrC using the TigrFam TIGR02912 profile are also listed.

Supplementary Table 1 is provided as separate Excel file.

Supplementary Table 2 | Query sequences used for diamond blastp. Sequences without reference are homologues present in the genomes of query sequences.

Protein	***Subunit***	***Organism***	***Genome ID***	***Accession number***	***Ref.***
Dissimilatory sulfite reductases	DsrM	*Desulfovibrio vulgaris* str. Hildenborough	GCF_000195755.1	YP_010509.1	[19]
	DsrK	*Desulfovibrio vulgaris* str. Hildenborough	GCF_000195755.1	YP_010508.1	[19]
	DsrJ	*Desulfovibrio vulgaris* str. Hildenborough	GCF_000195755.1	YP_010507.1	[19]
	DsrO	*Desulfovibrio vulgaris* str. Hildenborough	GCF_000195755.1	YP_010506.1	[19]
	DsrP	*Desulfovibrio vulgaris* str. Hildenborough	GCF_000195755.1	YP_010505.1	[19]
	DsrM	*Archaeoglobus fulgidus* DSM 4304	GCF_000008665.1	WP_010878008.1	[20]
	DsrK	*Archaeoglobus fulgidus* DSM 4304	GCF_000008665.1	WP_010878009.1	[20]
	DsrM	*Archaeoglobus fulgidus* DSM 4304	GCF_000008665.1	WP_010878052.1	[20]
	DsrK	*Archaeoglobus fulgidus* DSM 4304	GCF_000008665.1	WP_010878051.1	[20]
	DsrK	*Archaeoglobus fulgidus* DSM 4304	GCF_000008665.1	WP_010878050.1	[20]
	DsrM	*Archaeoglobus fulgidus* DSM 4304	GCF_000008665.1	WP_010878053.1	[20]
	DsrK	*Archaeoglobus fulgidus* DSM 4304	GCF_000008665.1	WP_010878054.1	[20]
	DsrJ	*Archaeoglobus fulgidus* DSM 4304	GCF_000008665.1	WP_048064247.1	[20]
	DsrO	*Archaeoglobus fulgidus* DSM 4304	GCF_000008665.1	WP_010878006.1	[20]
	DsrP	*Archaeoglobus fulgidus* DSM 4304	GCF_000008665.1	WP_010878007.1	[20]
	DsrM	*Caldivirga maquilingensis* IC-167	GCF_000018305.1	WP_012185906.1	[20]
	DsrK	*Caldivirga maquilingensis* IC-167	GCF_000018305.1	WP_083755284.1	[20]
	DsrM	*Caldivirga maquilingensis* IC-167	GCF_000018305.1	WP_012185636.1	
	DsrK	*Caldivirga maquilingensis* IC-167	GCF_000018305.1	WP_012185635.1	
	DsrU	*Chlorobaculum tepidum* TLS	GCF_000006985.1	NP_663120.1	[21]
	DsrV	*Chlorobaculum tepidum* TLS	GCF_000006985.1	NP_663113.1	[21]
	DsrW	*Chlorobaculum tepidum* TLS	GCF_000006985.1	NP_663112.1	[21]
	DsrR	*Allochromatium vinosum* DSM 180	GCF_000025485.1	WP_043796041.1	[22]
	DsrS	*Allochromatium vinosum* DSM 180	GCF_000025485.1	WP_012970478.1	[21]
	DsrN	*Chlorobaculum tepidum* TLS	GCF_000006985.1	NP_663124.1	[21]
	DsrN	*Desulfobacter vibrioformis* DSM 8776	GCF_000745975.1	WP_035235562.1	[23]
	DsrN	*Allochromatium vinosum* DSM 180	GCF_000025485.1	WP_012970476.1	[22]
Cobyrinic acid a,c-diamide synthase	CobB	*Ensifer adhaerens*	GCF_000697965.2	WP_034788296.1	[24]
	CbiA	*Salmonella typhimurium* LT2	GCF_000006945.2	NP_460980.1	[25]
	CbiA	*Bacillus megaterium*	GCF_000832985.1	WP_034651560.1	[26]
Coenzyme F_{430} biosynthesis	CfbB	*Methanosarcina acetivorans* C2A	GCA_000007345.1	AAM06981.1	[27]
Heterodisulfide reductases	HdrED-like	*Archaeoglobus fulgidus* DSM 4304	GCF_000008665.1	WP_010878258.1	
	HdrE	*Methanosarcina acetivorans* C2A	GCA_000007345.1	AAM04127.1	[28]
	HdrD	*Methanosarcina acetivorans* C2A	GCA_000007345.1	AAM04128.1	[28]
	HdrD	*Methanosarcina acetivorans* C2A	GCA_000007345.1	AAM03970.1	
	HdrD-like	*Methanosarcina acetivorans* C2A	GCA_000007345.1	AAM07964.1	
	HdrF	*Desulfotomaculum reducens* MI-1	GCF_000016165.1	WP_011878115.1	[29]

Supplementary Table 2 | Query sequences used for diamond blastp. Continued.

Protein	*Subunit*	*Organism*	*Genome ID*	*Accession number*	*Ref.*
High-molecular weight cytochrome complex	HmcA	*Desulfovibrio vulgaris* str. Hildenborough	GCF_000195755.1	YP_009759.1	[20, 30]
	HmcB	*Desulfovibrio vulgaris* str. Hildenborough	GCF_000195755.1	YP_009758.1	[20, 30]
	HmcC	*Desulfovibrio vulgaris* str. Hildenborough	GCF_000195755.1	YP_009757.1	[20, 30]
	HmcD	*Desulfovibrio vulgaris* str. Hildenborough	GCF_000195755.1	YP_009756.1	[20, 30]
	HmcE	*Desulfovibrio vulgaris* str. Hildenborough	GCF_000195755.1	YP_009755.1	[20, 30]
	HmcF	*Desulfovibrio vulgaris* str. Hildenborough	GCF_000195755.1	YP_009754.1	[20, 30]
Tetraheme membrane cytochrome complex	TmcA	*Desulfovibrio vulgaris* str. Hildenborough	GCF_000195755.1	YP_009487.1	[20, 30]
	TmcB	*Desulfovibrio vulgaris* str. Hildenborough	GCF_000195755.1	YP_009488.1	[20, 30]
	TmcC	*Desulfovibrio vulgaris* str. Hildenborough	GCF_000195755.1	YP_009489.1	[20, 30]
	TmcD	*Desulfovibrio vulgaris* str. Hildenborough	GCF_000195755.1	YP_009490.2	[20, 30]
Octaheme cytochrome complex	OhcA	*Desulfovibrio vulgaris* str. Hildenborough	GCF_000195755.1	YP_012353.1	[20, 30]
	OhcB	*Desulfovibrio vulgaris* str. Hildenborough	GCF_000195755.1	YP_012354.1	[20, 30]
	OhcC	*Desulfovibrio vulgaris* str. Hildenborough	GCF_000195755.1	YP_012355.1	[20, 30]
Nitrate reductases and chaperones	NarG	*Escherichia coli* str. K-12 substr. MG1655	GCF_000005845.2	NP_415742.1	[31]
	NarI	*Escherichia coli* str. K-12 substr. MG1655	GCF_000005845.2	NP_415745.1	[31]
	NarJ	*Escherichia coli* str. K-12 substr. MG1655	GCF_000005845.2	NP_415744.1	[31]
	NarH	*Escherichia coli* str. K-12 substr. MG1655	GCF_000005845.2	NP_415743.1	[31]
	NarV	*Escherichia coli* str. K-12 substr. MG1655	GCF_000005845.2	NP_415982.1	[32]
	NarW	*Escherichia coli* str. K-12 substr. MG1655	GCF_000005845.2	NP_415983.1	[32]
	NarY	*Escherichia coli* str. K-12 substr. MG1655	GCF_000005845.2	NP_415984.1	[32]
	NarZ	*Escherichia coli* str. K-12 substr. MG1655	GCF_000005845.2	NP_415985.1	[32]

Supplementary Table 3 | Redundancy reduction using a 90% global identity cut-off. The total number of identified sequences, unique sequences, sequences after the redundancy filtering used in phylogenetic analysis, and sequences belonging to complete genomes is given.

Protein	*Identified sequences*	*Identified unique sequences*	*Sequences after 90% identity redundancy reduction*	*Sequences from complete genomes*
DsrA	1884	1730	1052	131
DsrB	1841	1679	962	118
DsrC	1921	1708	861	117
DsrD	918	839	545	62
DsrE	1027	899	471	70
DsrF	814	730	415	58
DsrH	795	707	454	63
DsrJ	1477	1340	981	114
DsrK	1723	1595	1001	127
DsrL	887	814	517	56
DsrM	1753	1624	1122	139
DsrO	1557	1434	1022	109
DsrP	1605	1479	1003	109
DsrT	802	748	581	69
DsrN	8000	5396	3241	716
DsrA DsrB AsrC	4906	3673	2099	334
AprA	2272	1570	791	87
AprB	2190	1606	728	92
QmoA	1043	1062	684	80
QmoB	1347	1374	964	88
QmoC	973	998	720	56
AprA SdhA	2353	1651	872	168
QmoA HdrA	1057	1076	698	94
QmoB HdrA	1361	1388	978	102

Supplementary Table 4 | Alignment lengths before and after trimming. The number of positions of each multiple sequence alignment before and after trimming using a 95% gap threshold is given for the two sequence sets.

	Full diversity		***Complete genomes***	
Protein	**Alignment length**	**Alignment length after trimming**	**Alignment length**	**Alignment length after trimming**
DsrA	708	515	574	512
DsrB	620	403	469	421
DsrC	223	119	128	120
DsrD	312	98	98	90
DsrE	175	130	146	133
DsrF	186	148	146	145
DsrH	126	104	103	103
DsrJ	670	238	297	220
DsrK	874	633	714	633
DsrL	1099	712	736	709
DsrM	807	414	492	389
DsrO	590	345	365	317
DsrP	648	455	525	458
DsrT	439	232	225	211
DsrN	2799	647	1471	568
DsrA DsrB AsrC	838	515	649	552
AprA	994	740	751	726
AprB	355	197	201	191
QmoA	805	443	474	432
QmoB	1989	976	992	942
QmoC	1213	516	599	513
AprA SdhA	1190	791	1033	837
QmoA HdrA	1085	444	881	673
QmoB HdrA	2029	977	1086	947

Supplementary Table 5 | Best model for each Dsr protein set according to BIC. Amino acid substitution model of the phylogenies of Dsr proteins present in only complete genomes and phylogenies of the sequences set covering the full diversity including metagenome-derived genomic assemblies.

Protein	***Full diversity, 90% redundancy reduction***	***Complete genomes***
DsrA	LG+I+G4	LG+I+G4
DsrB	LG+I+G4	LG+I+G4
DsrC	LG+I+G4	LG+G4
DsrD	LG+F+G4	WAG+I+G4
DsrE	LG+I+G4	LG+I+G4
DsrF	LG+G4	LG+G4
DsrH	LG+F+G4	LG+I+G4
DsrL	LG+I+G4	LG+I+G4
DsrM	LG+F+I+G4	LG+F+I+G4
DsrK	LG+I+G4	LG+I+G4
DsrJ	WAG+I+G4	WAG+I+G4
DsrO	WAG+I+G4	WAG+I+G4
DsrP	LG+F+I+G4	LG+F+G4
DsrT	LG+G4	LG+G4
DsrN	LG+F+G4	LG+I+G4
DsrA DsrB AsrC	LG+I+G4	LG+I+G4
AprA	LG+F+I+G4	LG+F+I+G4
AprB	WAG+I+G4	WAG+I+G4
QmoA	LG+I+G4	LG+I+G4
QmoB	LG+I+G4	LG+I+G4
QmoC	LG+I+G4	LG+F+I+G4
AprA SdhA	LG+F+I+G4	LG+F+I+G4
QmoA HdrA	LG+I+G4	LG+I+G4
QmoB HdrA	LG+I+G4	LG+I+G4

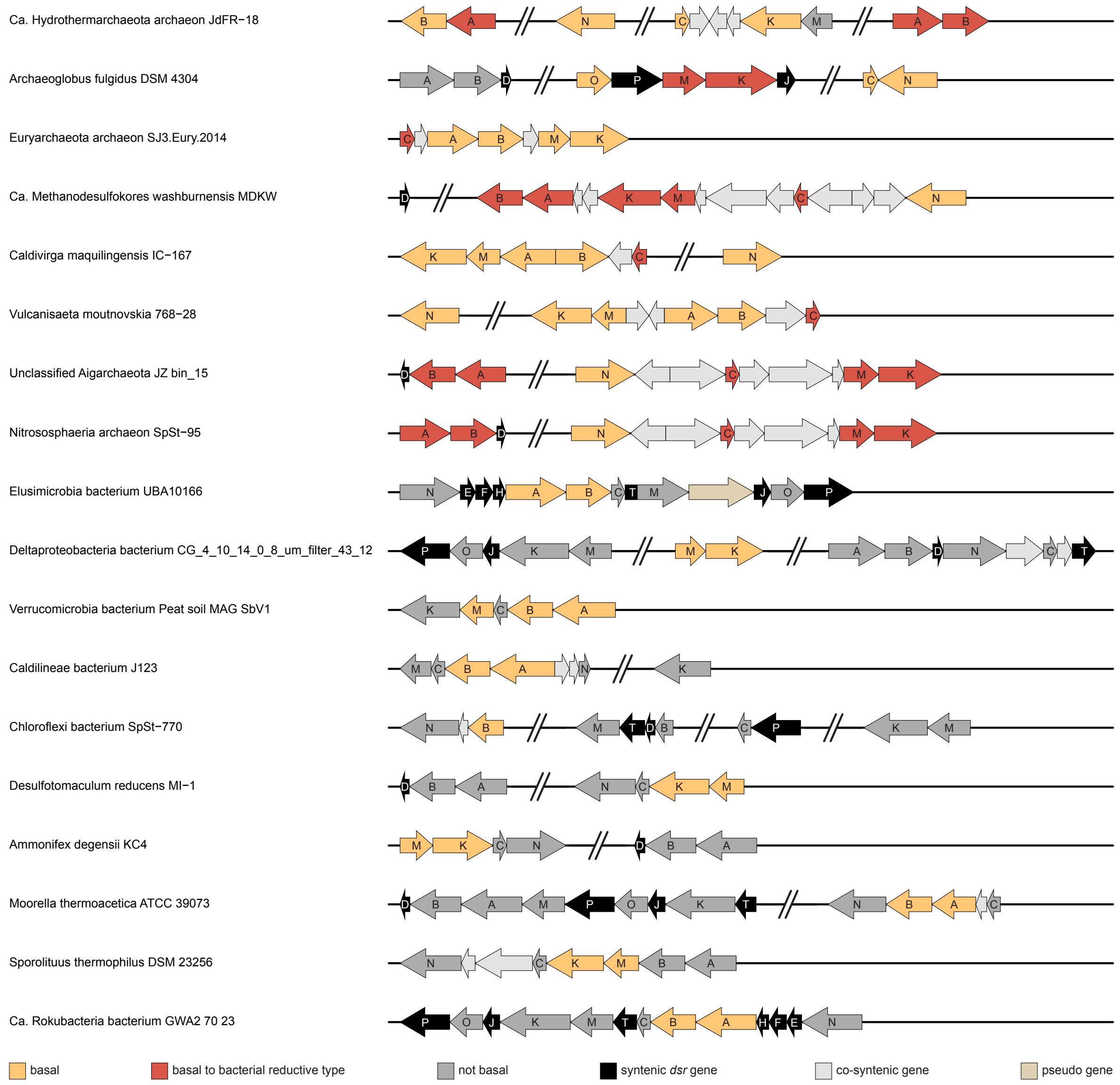

Supplementary Fig. 1 | Genomic neighborhood of genes encoding for Dsr proteins branching basal in the Dsr phylogenies. Genes whose protein sequences branch in basal clades in the corresponding phylogenies are colored in orange, the ones at the base of the bacterial reductive-type clade in red, the remaining sequences located within non-basal clades in dark grey. Phylogenies with low resolution (DsrD; DsrPJT) or type-specific proteins (DsrE/DsrL) were not classifiable regarding basal clades, and only the presence is indicated in black. Co-syntenic *non-dsr* genes are indicated with light gray and pseudo genes in light brown. Letters in genomic colocalizations indicate the subunit of Dsr proteins.

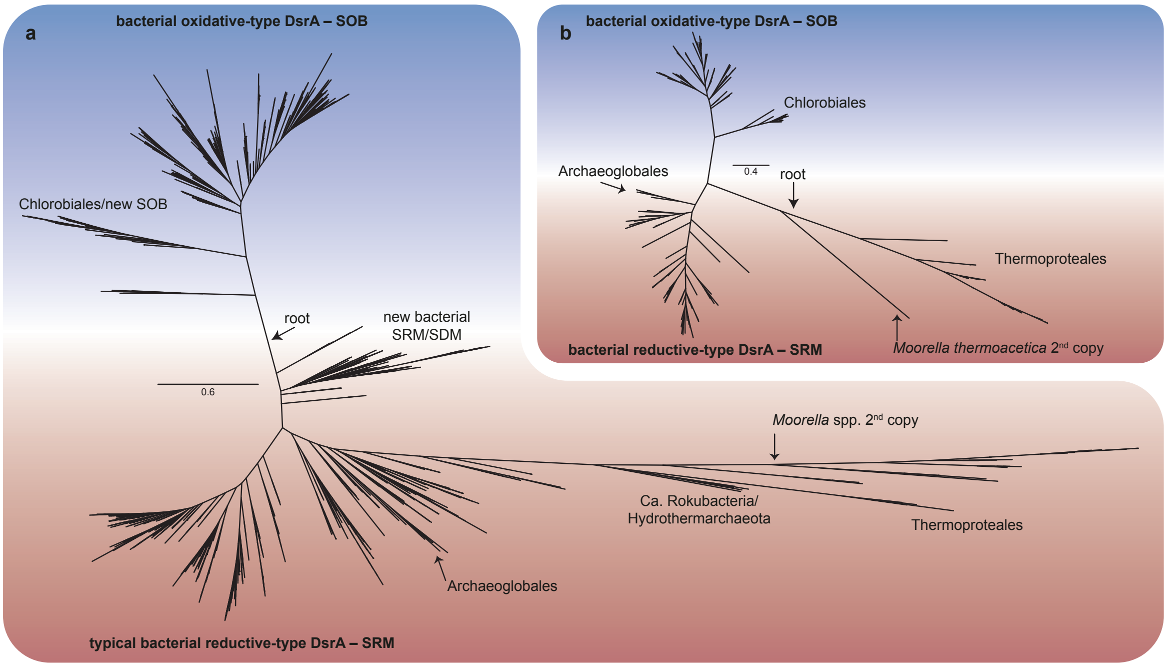

Supplementary Fig. 2 | Phylogenetic reconstruction sulfite reductases a) of DsrA proteins from (meta)genomes and b) of DsrA proteins from complete genomes. (models for phylogenetic reconstructions: LG+I+G4).

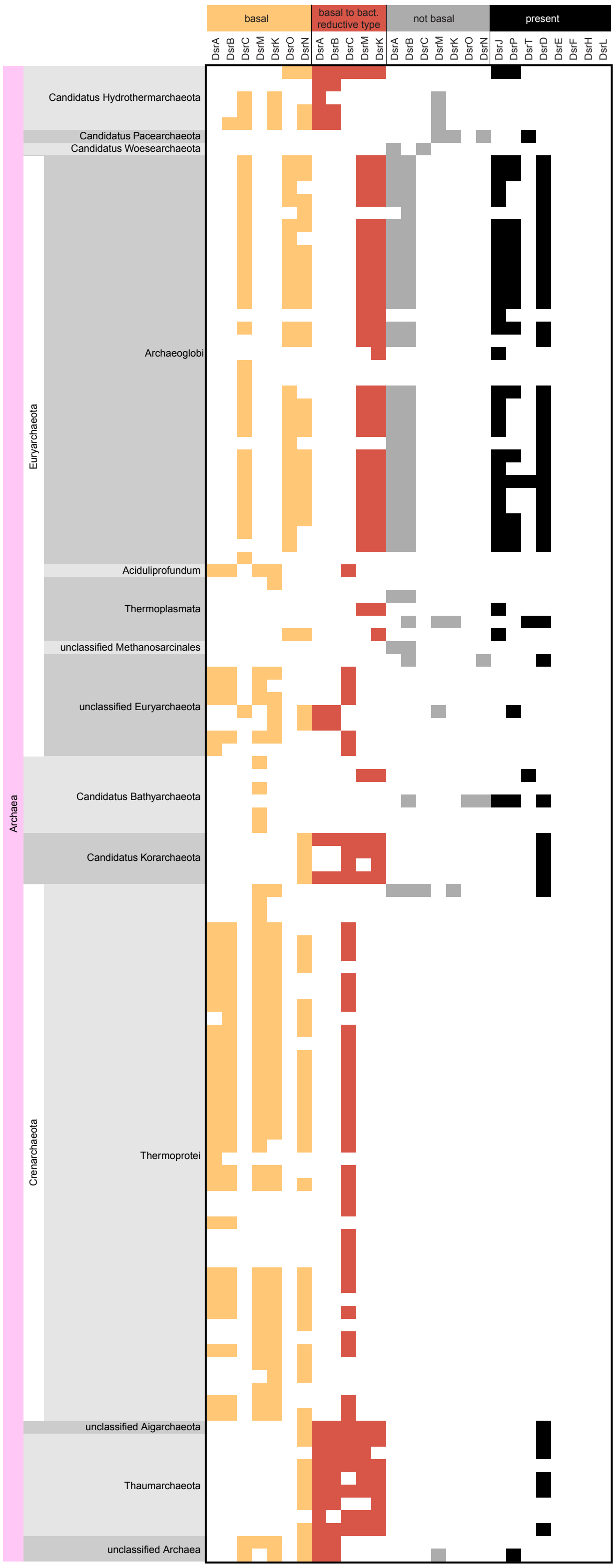

Supplementary Fig. 3 | Distribution and phylogenetic clade classification of Dsr proteins across Archaea. Color code according to Supplementary Figure 1.

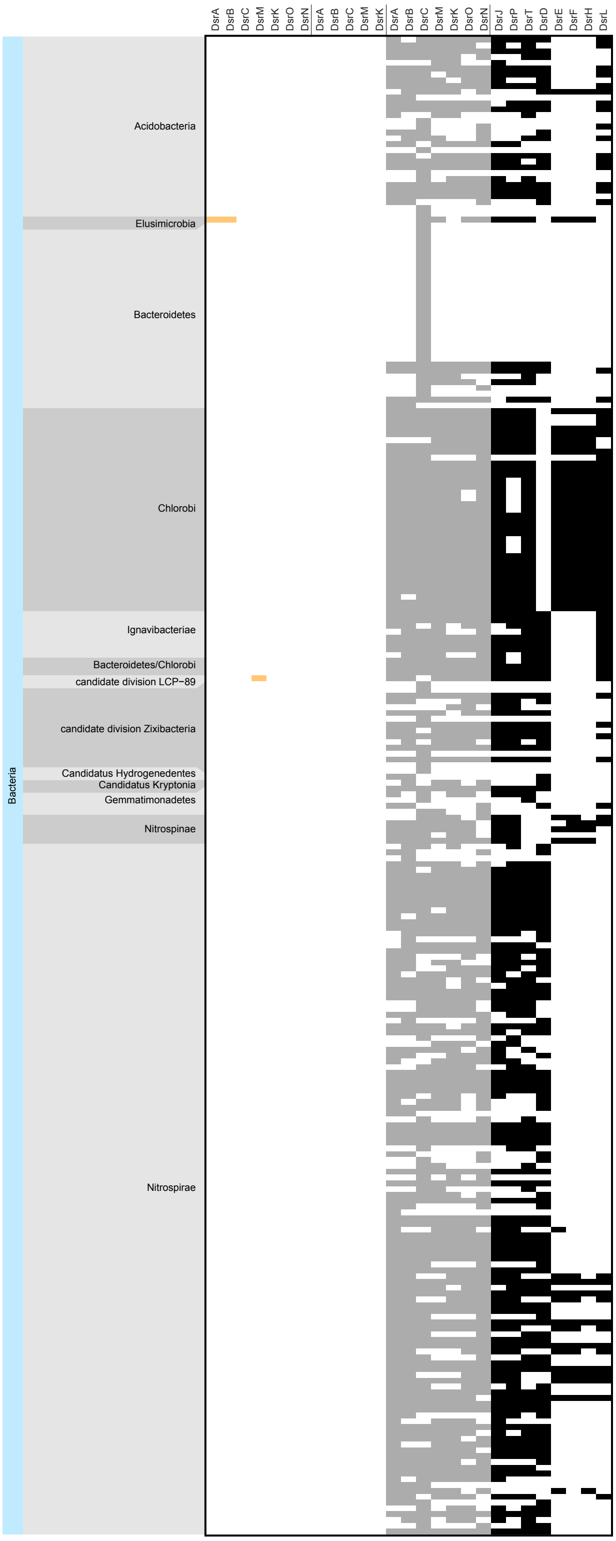

Supplementary Fig. 4 | Distribution and phylogenetic clade classification of Dsr proteins across *Acidobacteria*, *Elusimicrobia*, FCB group, *Nitrospinae*, and *Nitrospirae*. Color code according to Supplementary Figure 1.

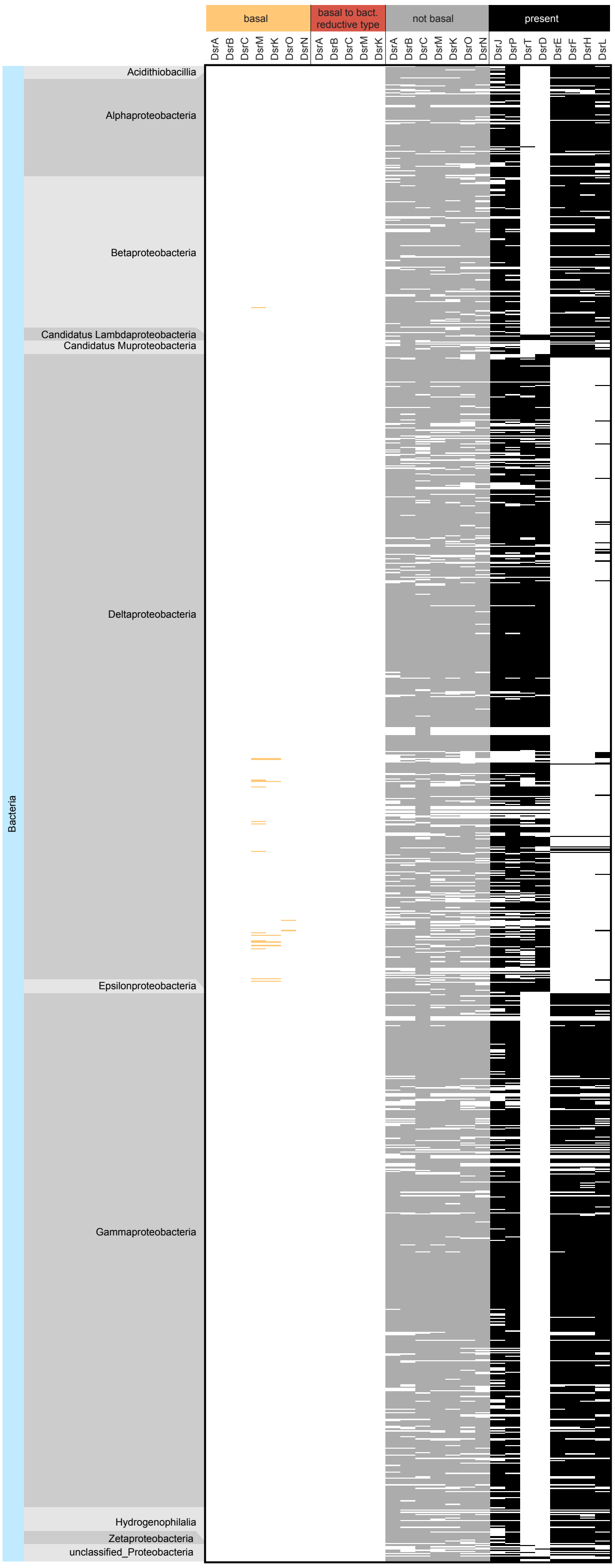

Supplementary Fig. 5 | Distribution and phylogenetic clade classification of Dsr proteins across *Proteobacteria*. Color code according to Supplementary Figure 1.

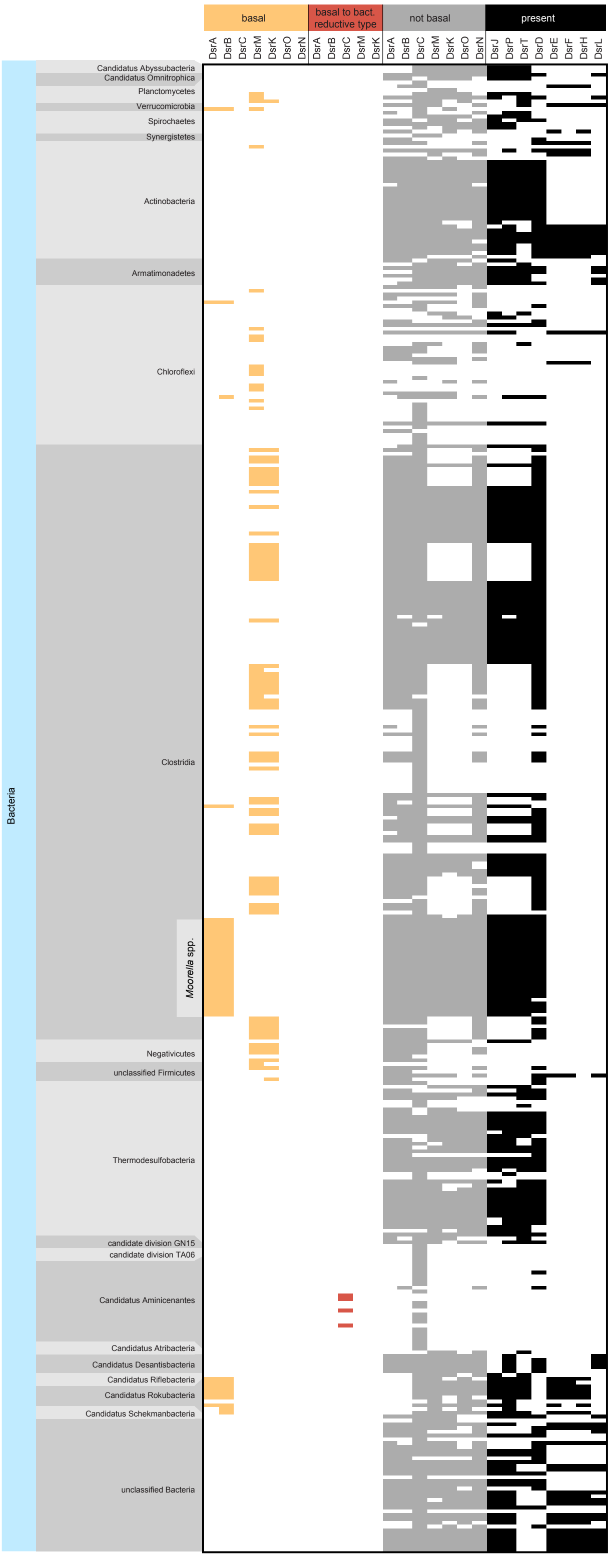

Supplementary Fig. 6 | Distribution and phylogenetic clade classification of Dsr proteins across PVC group, *Spirochaetes*, *Synergistetes*, *Actinobacteria*, *Armatimonadetes*, *Chloroflexi*, *Firmicutes*, *Thermodesulfobacteria*, and bacterial candidate phyla. Color code according to Supplementary Figure 1.

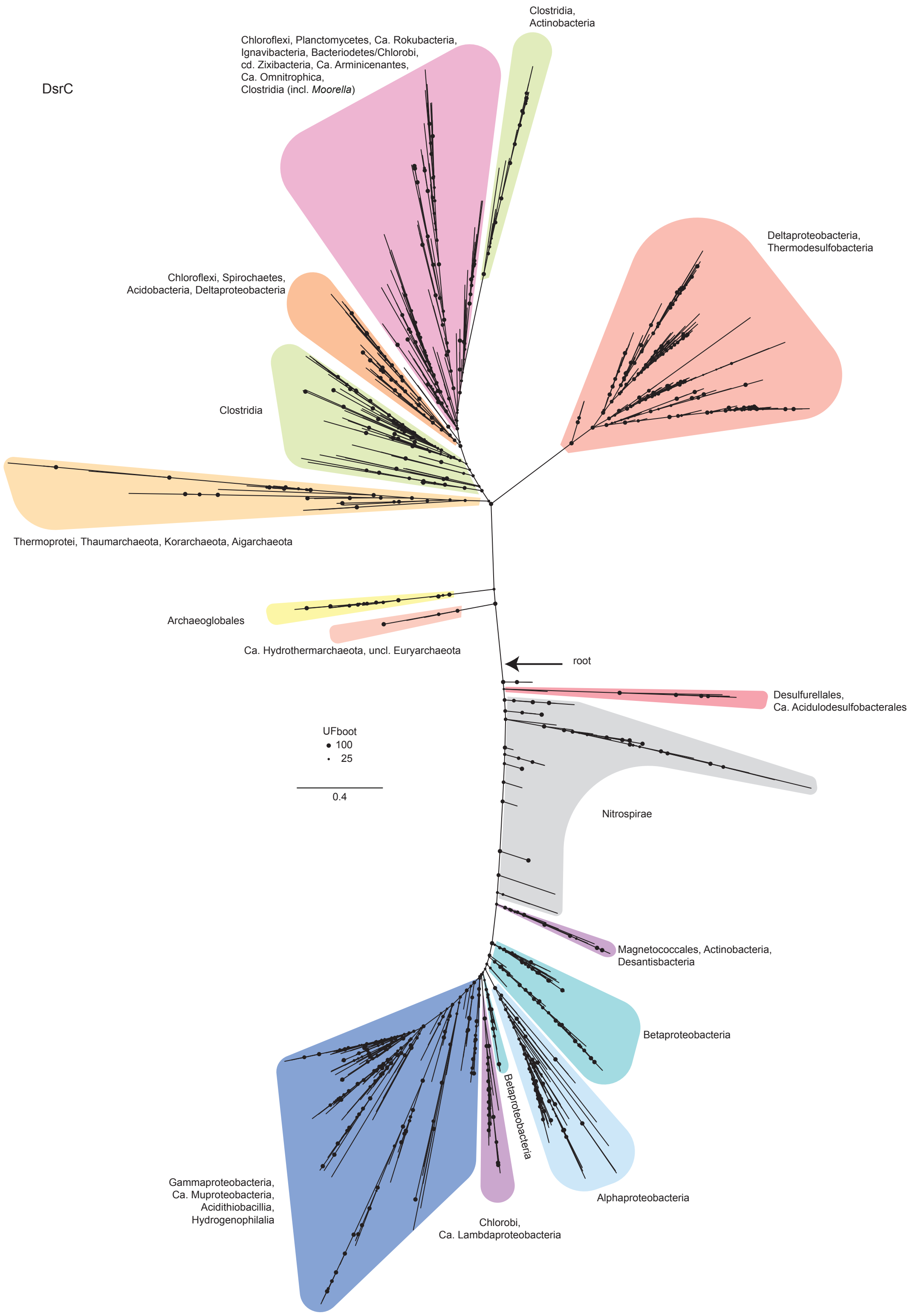

Supplementary Fig. 7 | Maximum likelihood phylogeny of DsrC proteins (model LG+I+G4). Black circles indicate ultrafast bootstrap values (UFboot).

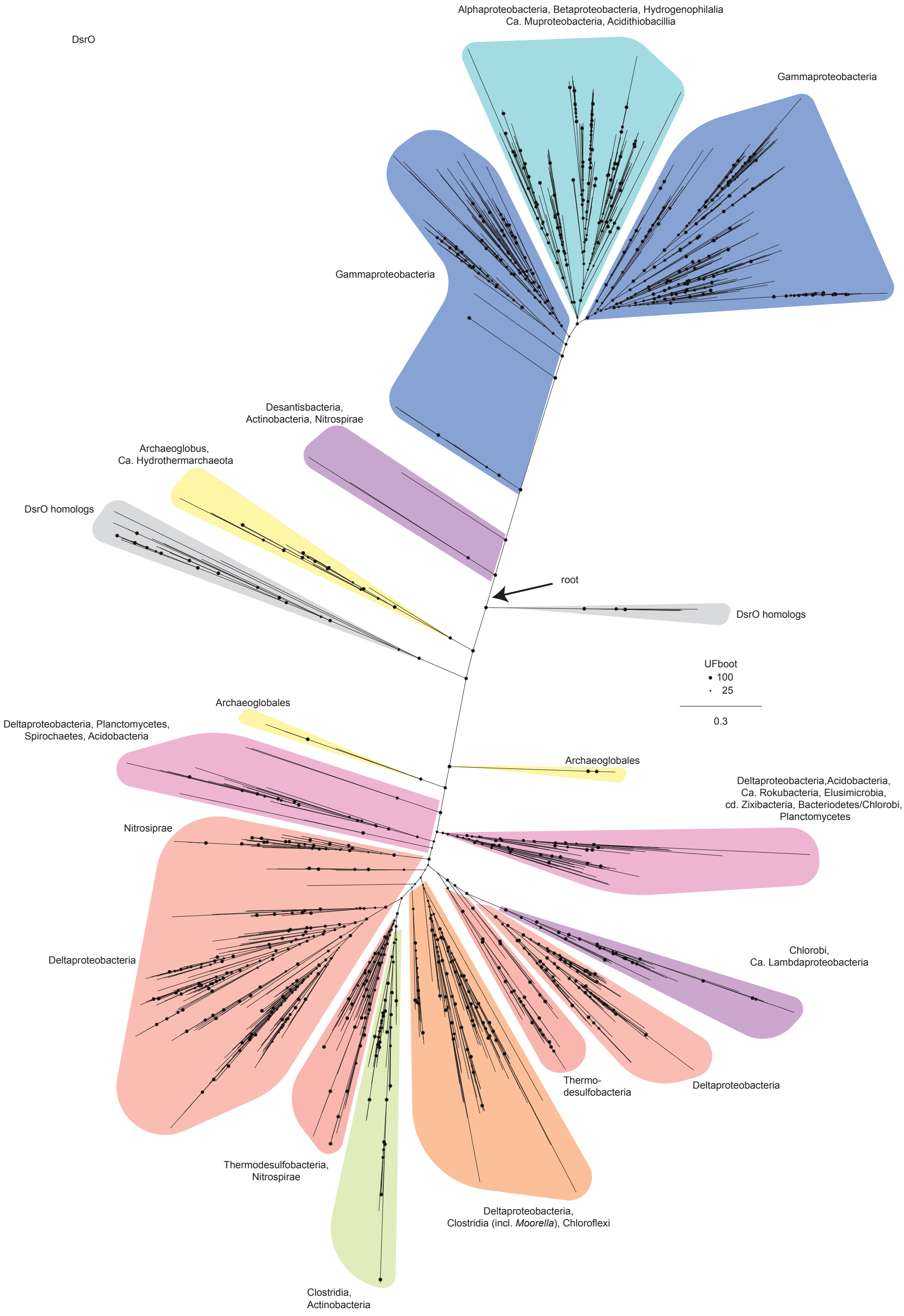

Supplementary Fig. 8 | Maximum likelihood phylogeny of DsrO proteins (model LG+I+G4). Black circles indicate ultrafast bootstrap values.

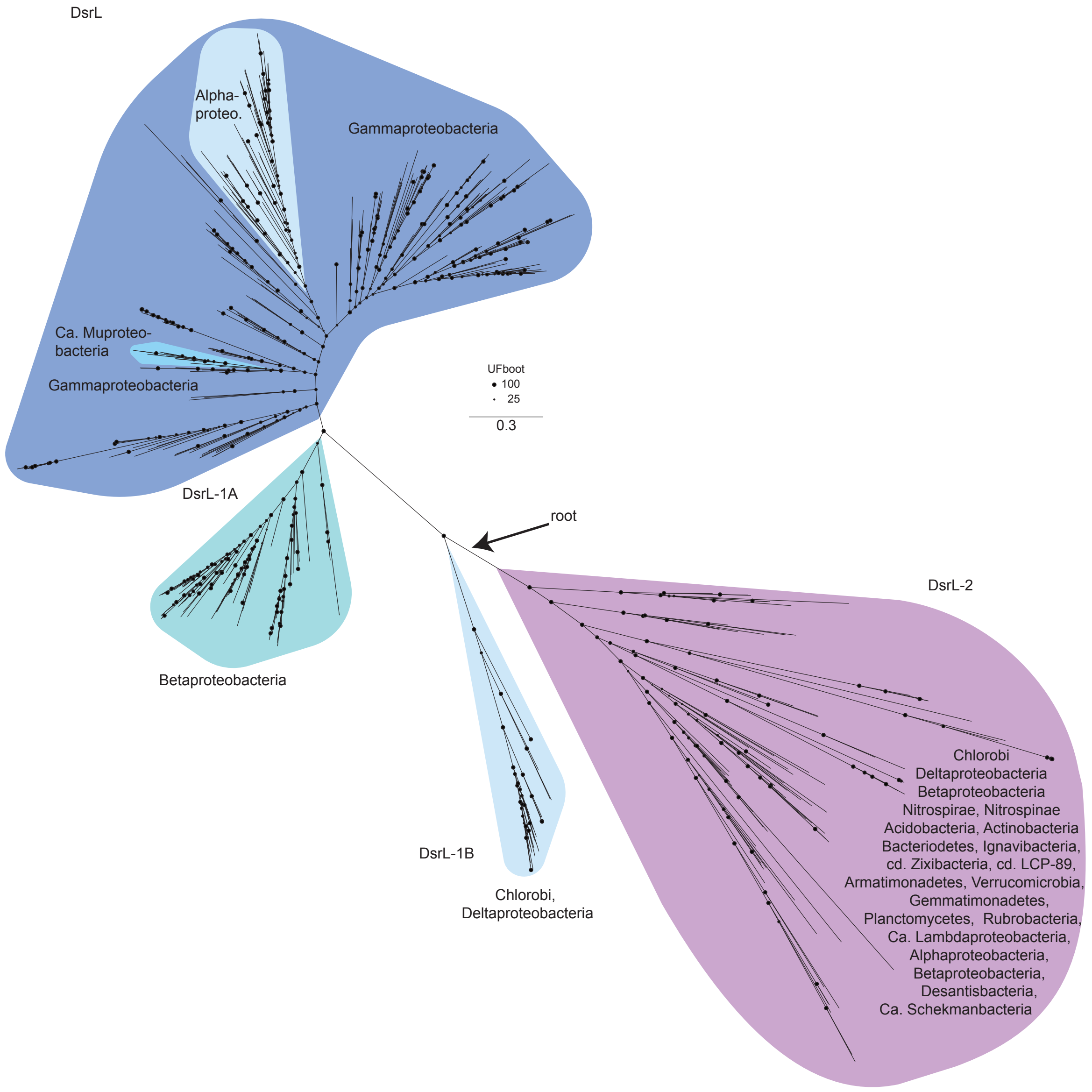

Supplementary Fig. 9 | Maximum likelihood phylogeny of DsrL proteins (model LG+I+G4). Black circles indicate ultrafast bootstrap values. DsrL type classification of DsrL-1A, DsrL-1B, and DsrL-2 according to [33].

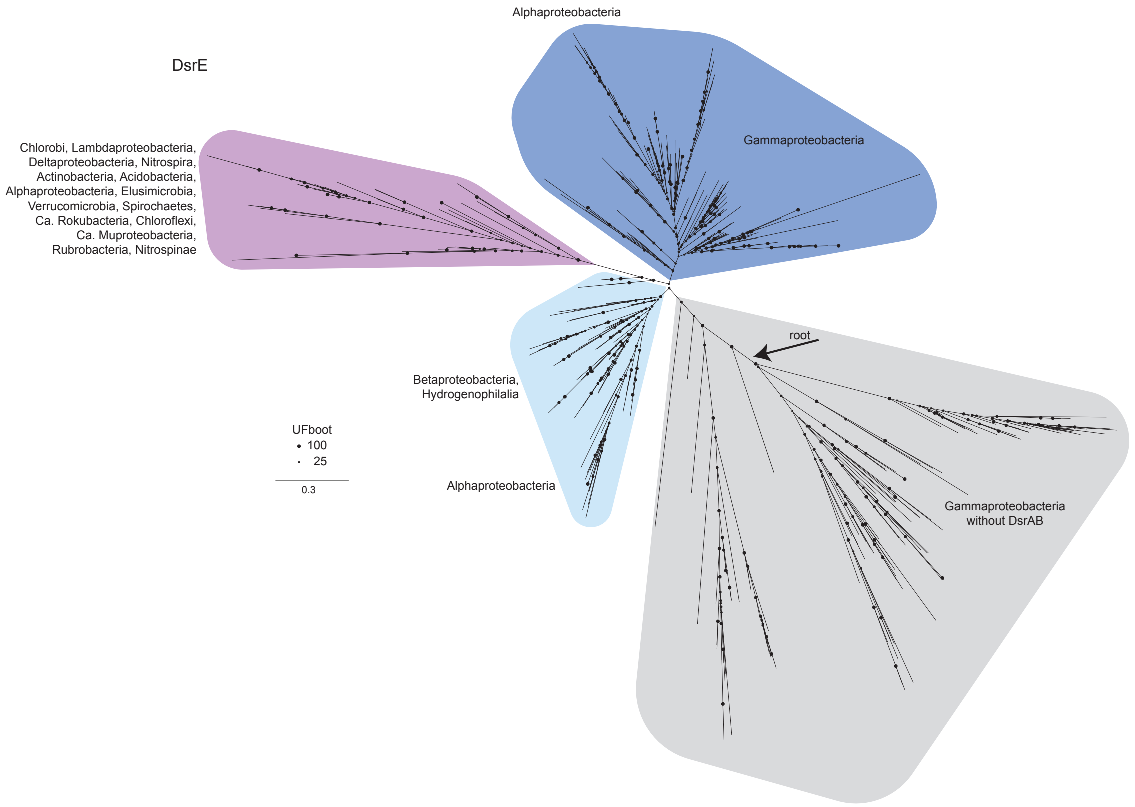

Supplementary Fig. 10 | Maximum likelihood phylogeny of DsrE proteins (model LG+I+G4). Black circles indicate ultrafast bootstrap values.

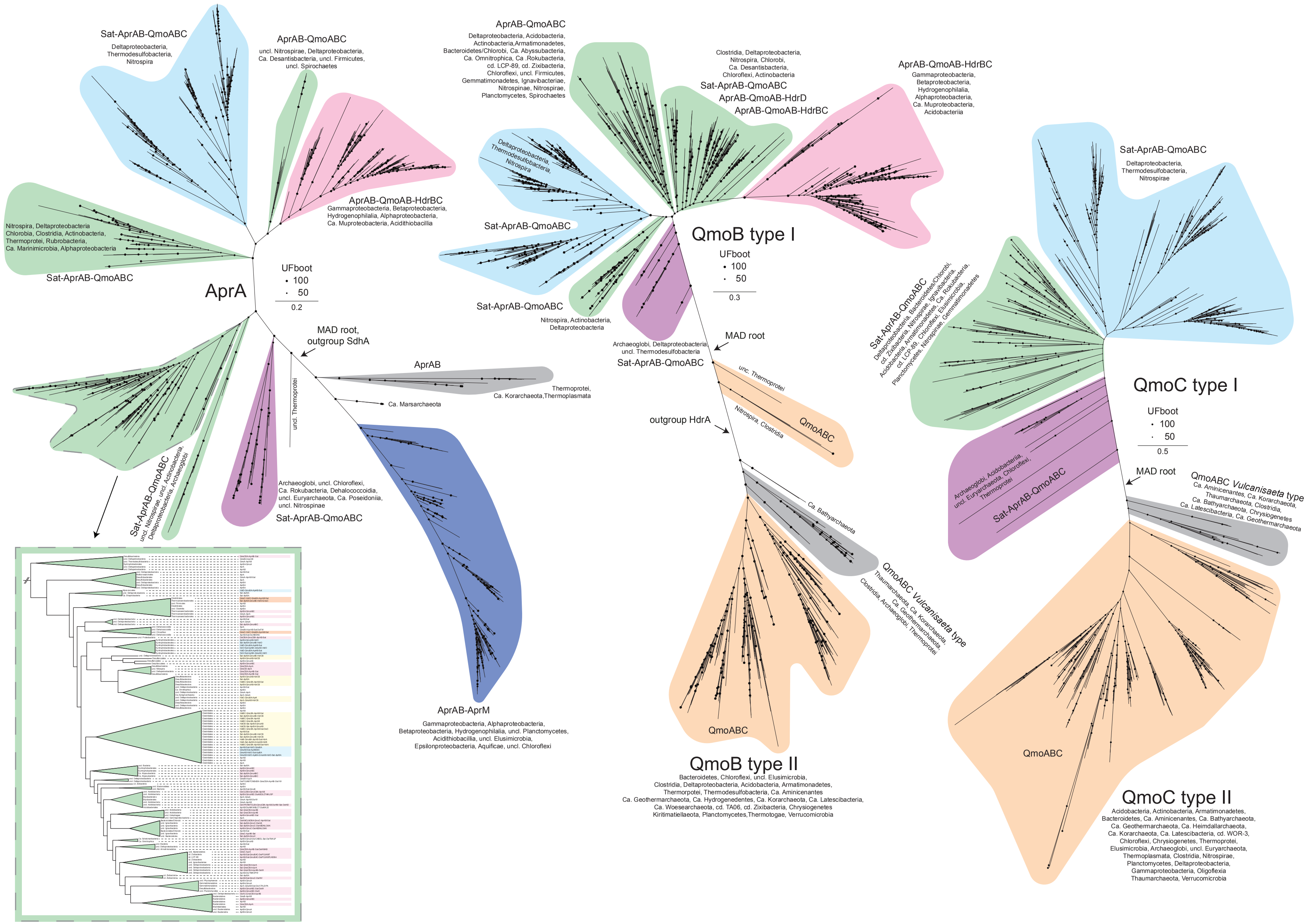

AprA
UFboot
100
50
0.2
Sat-AprAB-QmoABC
Deltaproteobacteria, Thermodesulfobacteria, Nitrospira
AprAB-QmoABC
uncl. Nitrospirae, Deltaproteobacteria, Ca. Desantisbacteria, uncl. Firmicutes, uncl. Spirochaetes
AprAB-QmoAB-HdrBC
Gammaproteobacteria, Betaproteobacteria, Hydrogenophilalia, Alphaproteobacteria, Ca. Muproteobacteria, Acidithiobacillia
Nitrospira, Deltaproteobacteria Chlorobia, Clostridia, Actinobacteria, Thermoprotei, Rubrobacteria, Ca. Marinimicrobia, Alphaproteobacteria
Sat-AprAB-QmoABC
MAD root, outgroup SdhA
AprAB
Thermoprotei, Ca. Korarchaeota,Thermoplasmata
Ca. Marsarchaeota
uncl. Thermoprotei
Sat-AprAB-QmoABC
uncl. Nitrospirae, uncl. Actinobacteria, Deltaproteobacteria, Archaeoglobi
Archaeoglobi, uncl. Chloroflexi, Ca. Rokubacteria, Dehalococcoidia, uncl. Euryarchaeota, Ca. Poseidoniia, uncl. Nitrospinae
Sat-AprAB-QmoABC
AprAB-AprM
Gammaproteobacteria, Alphaproteobacteria, Betaproteobacteria, Hydrogenophilalia, uncl. Planctomycetes, Acidithiobacillia, uncl. Elusimicrobia, Epsilonproteobacteria, Aquificae, uncl. Chloroflexi
QmoB type I
UFboot
100
50
0.3
AprAB-QmoABC
Deltaproteobacteria, Acidobacteria, Actinobacteria,Armatimonadetes, Bacteroidetes/Chlorobi, Ca. Abyssubacteria, Ca. Omnitrophica, Ca .Rokubacteria, cd. LCP-89, cd. Zixibacteria, Chloroflexi, uncl. Firmicutes, Gemmatimonadetes, Ignavibacteriae, Nitrospinae, Nitrospirae, Planctomycetes, Spirochaetes
Clostridia, Deltaproteobacteria, Nitrospira, Chlorobi, Ca. Desantisbacteria, Chloroflexi, Actinobacteria
Sat-AprAB-QmoABC
AprAB-QmoAB-HdrD
AprAB-QmoAB-HdrBC
AprAB-QmoAB-HdrBC
Gammaproteobacteria, Betaproteobacteria, Hydrogenophilalia, Alphaproteobacteria, Ca. Muproteobacteria, Acidobacteriia
Deltaproteobacteria, Thermodesulfobacteria, Nitrospira
Sat-AprAB-QmoABC
Sat-AprAB-QmoABC
Nitrospira, Actinobacteria, Deltaproteobacteria
Archaeoglobi, Deltaproteobacteria, uncl. Thermodesulfobacteria
Sat-AprAB-QmoABC
MAD root
unc. Thermoprotei
QmoABC
Nitrospira, Clostridia
outgroup HdrA
Ca. Bathyarchaeota
QmoABC Vulcanisaeta type
Thaumarchaeota, Ca. Korarchaeota, Ca. Geothermarchaeota, Clostridia, Archaeoglobi, Thermoprotei
QmoABC
QmoB type II
Bacteroidetes, Chloroflexi, uncl. Elusimicrobia, Clostridia, Deltaproteobacteria, Acidobacteria, Armatimonadetes, Thermorpotei, Thermodesulfobacteria, Ca. Aminicenantes
Ca. Geothermarchaeota, Ca. Hydrogenedentes, Ca. Korarchaeota, Ca. Latescibacteria, Ca. Woesearchaeota, cd. TA06, cd. Zixibacteria, Chrysiogenetes
Kiritimatiellaeota, Planctomycetes,Thermotogae, Verrucomicrobia
Sat-AprAB-QmoABC
Deltaproteobacteria, Thermodesulfobacteria, Nitrospirae
Sat-AprAB-QmoABC
Deltaproteobacteria, Bacteroidetes/Chlorobi, cd. Zixibacteria, Nitrospirae, Ignavibacteria, Acidobacteria, Armatimonadetes, Ca. Rokubacteria, cd. LCP-89, Chloroflexi, Elusimicrobia, Planctomycetes, Nitrospinae, Gemmatimonadetes
QmoC type I
UFboot
100
50
0.5
MAD root
Archaeoglobi, Acidobacteria, uncl. Euryarchaeota, Chloroflexi, Thermoprotei
Sat-AprAB-QmoABC
QmoABC Vulcanisaeta type
Ca. Aminicenantes, Ca. Korarchaeota, Thaumarchaeota, Clostridia, Ca. Bathyarchaeota, Chrysiogenetes, Ca. Latescibacteria, Ca. Geothermarchaeota
QmoABC
QmoC type II
Acidobacteria, Actinobacteria, Armatimonadetes, Bacteroidetes, Ca. Aminicenantes, Ca. Bathyarchaeota, Ca. Geothermarchaeota, Ca. Heimdallarchaeota, Ca. Korarchaeota, Ca. Latescibacteria, cd. WOR-3, Chloroflexi, Chrysiogenetes, Thermoprotei, Elusimicrobia, Archaeoglobi, uncl. Euryarchaeota, Thermoplasmata, Clostridia, Nitrospirae, Planctomycetes, Deltaproteobacteria, Gammaproteobacteria, Oligoflexia, Thaumarchaeota, Verrucomicrobia

Supplementary Fig. 11 | Maximum likelihood phylogenies of AprA, QmoB, and QmoC proteins (models for phylogenetic reconstructions: LG+I+G4). Black circles indicate ultrafast bootstrap values. The inset shows the detail syntenic distribution of the Qmo complex with the different arrangements QmoABC, QmoAB-HdrBC, and QmoAB-HdrD indicated in orange, blue, pink and yellow.

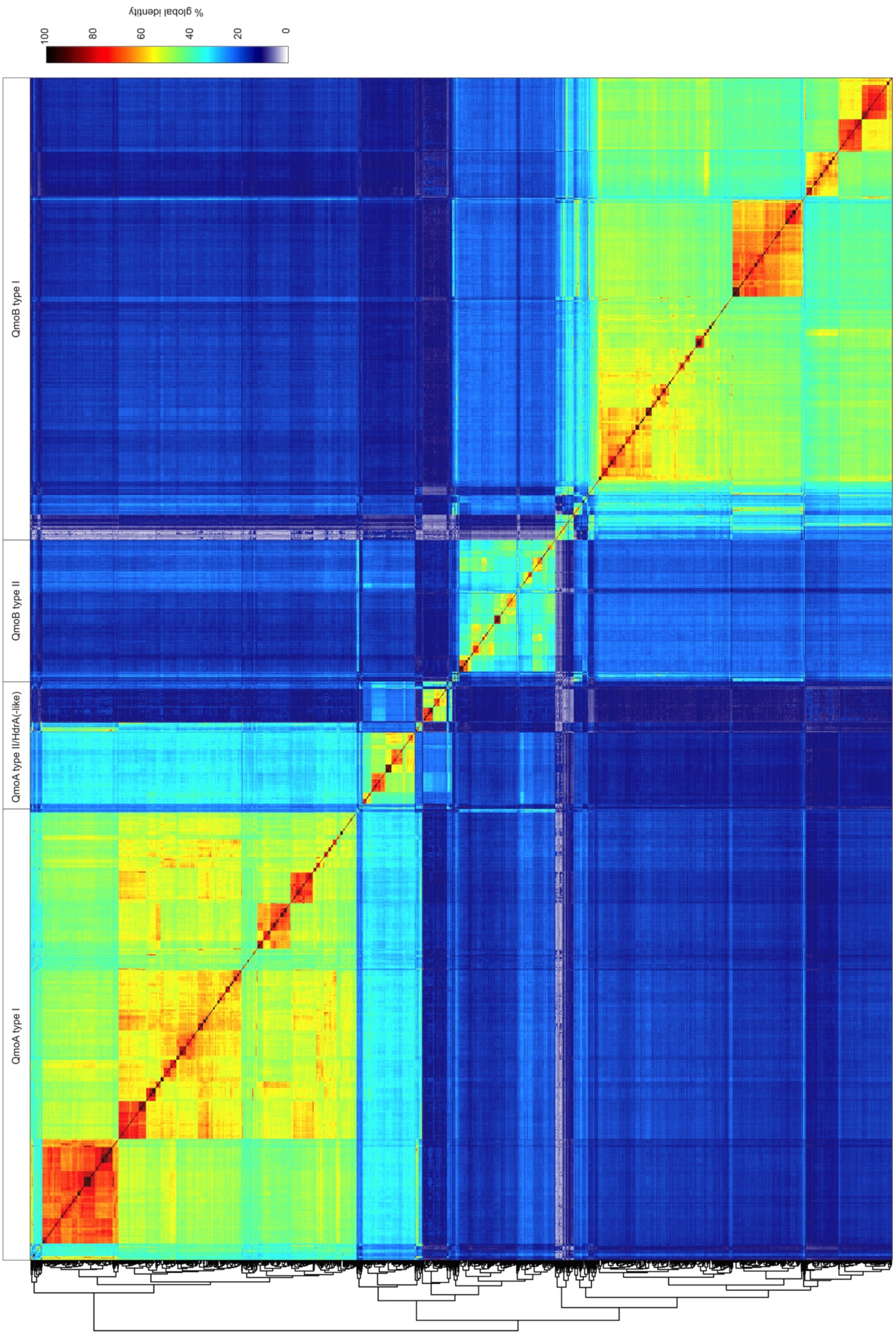

Supplementary Fig. 12 | Similarity of type I and type II QmoA, QmoB, and HdrA(-like) proteins. Global identities are represented by the colour code.

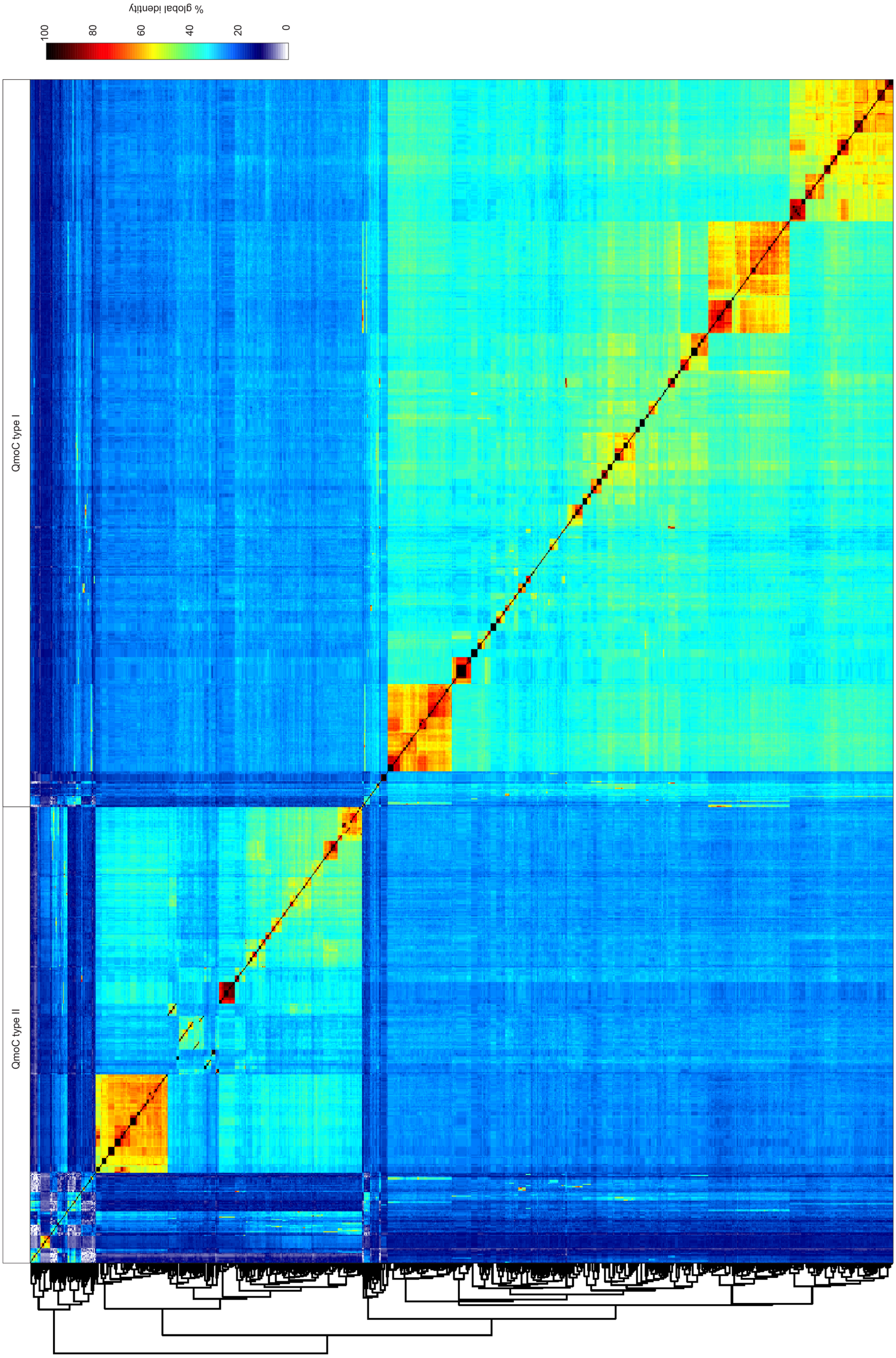

Supplementary Fig. 13 | Similarity of type I and type II QmoC proteins. Global identities are represented by the colour code.

Supplementary Data description

Available at doi.org/10.6084/m9.figshare.20766064

Supplementary Data 1 – FASTA files containing all amino acid sequences including the NCBI RefSeq/GenBank/IMG accession numbers per protein.

Supplementary Data 2 – Mapping table of selected sequences after 90 % global identity redundancy reduction to the corresponding protein sequence accession numbers, genome identifiers, and taxonomic affiliation.

Supplementary Data 3 – Trimmed multiple sequence alignments of protein sequences present in only complete genomes.

Supplementary Data 4 – Trimmed multiple sequence alignments of protein sequences covering the full diversity including metagenome-derived genomic assemblies.

Supplementary Data 5 – Phylogenetic reconstruction of protein sequences present in only complete genomes using ModelFinder[1] with best model selection.

Supplementary Data 6 – Phylogenetic reconstruction of protein sequences covering the full diversity including metagenome-derived genomic assemblies using ModelFinder[1] with best model selection.

Supplementary Data 7 – Phylogenetic reconstruction of protein sequences present in only complete genomes using the model LG+I+G4.

Supplementary Data 8 – Phylogenetic reconstruction of protein sequences covering the full diversity including metagenome-derived genomic assemblies using the model LG+I+G4.

References

1. Kalyaanamoorthy S, Minh BQ, Wong TKF, Von Haeseler A, Jermiin LS. ModelFinder: Fast model selection for accurate phylogenetic estimates. *Nat Methods* 2017; **14**: 587–589.
2. Le SQ, Gascuel O. An improved general amino acid replacement matrix. *Mol Biol Evol* 2008; **25**: 1307–1320.
3. Stockdreher Y, Venceslau SS, Josten M, Sahl HG, Pereira IAC, Dahl C. Cytoplasmic sulfurtransferases in the purple sulfur bacterium *Allochromatium vinosum*: Evidence for sulfur transfer from DsrEFH to DsrC. *PLoS One* 2012; **7**: e40785.
4. Müller A, Kjeldsen KU, Rattei T, Pester M, Loy A. Phylogenetic and environmental diversity of DsrAB-type dissimilatory (bi)sulfite reductases. *ISME J* 2015; **9**: 1152–1165.
5. Loy A, Duller S, Baranyi C, Mußmann M, Ott J, Sharon I, et al. Reverse dissimilatory sulfite reductase as phylogenetic marker for a subgroup of sulfur-oxidizing prokaryotes. *Environ Microbiol* 2009; **11**: 289–299.
6. Loy A, Duller S, Wagner M. Evolution and ecology of microbes dissimilating sulfur compounds: Insights from siroheme sulfite reductases. In: Dahl C, Friedrich CG (eds). *Microbial Sulfur Metabolism*. 2008. Springer, Heidelberg, pp 46–59.
7. Tria FDK, Landan G, Dagan T. Phylogenetic rooting using minimal ancestor deviation. *Nat Ecol Evol* 2017; **1**: 0193.
8. Anantharaman K, Hausmann B, Jungbluth SP, Kantor RS, Lavy A, Warren LA, et al. Expanded diversity of microbial groups that shape the dissimilatory sulfur cycle. *ISME J* 2018; **12**: 1715–1728.
9. Colman DR, Lindsay MR, Amenabar MJ, Fernandes-Martins MC, Roden ER, Boyd ES. Phylogenomic analysis of novel Diaforarchaea is consistent with sulfite but not sulfate reduction in volcanic environments on early Earth. *ISME J* 2020; **14**: 1316–1331.
10. Shen Y, Buick R, Canfield DE. Isotopic evidence for microbial sulphate reduction in the early Archaean era. *Nature* 2001; **410**: 77–81.
11. Sousa FL, Thiergart T, Landan G, Nelson-Sathi S, Pereira IAC, Allen JF, et al. Early bioenergetic evolution. *Philos Trans R Soc Lond B Biol Sci* 2013; **368**: 20130088.
12. Thauer RK, Jungermann K, Decker K. Energy conservation in chemotrophic anaerobic bacteria. *Bacteriol Rev* 1977; **41**: 100–180.
13. Duarte AG, Santos AA, Pereira IAC. Electron transfer between the QmoABC membrane complex and adenosine 5′-phosphosulfate reductase. *Biochim Biophys Acta - Bioenerg* 2016; **1857**: 380–386.
14. Chernyh NA, Neukirchen S, Frolov EN, Sousa FL, Miroshnichenko ML, Merkel AY, et al. Dissimilatory sulfate reduction in the archaeon '*Candidatus* Vulcanisaeta moutnovskia' sheds light on the evolution of sulfur metabolism. *Nat Microbiol* 2020; **5**: 1428–1438.
15. Ramos AR, Keller KL, Wall JD, Pereira IAC. The membrane QmoABC complex interacts directly with the dissimilatory adenosine 5′-phosphosulfate reductase in sulfate reducing bacteria. *Front Microbiol* 2012; **3**: 1–10.
16. Pires RH, Lourenço AI, Morais F, Teixeira M, Xavier A V., Saraiva LM, et al. A novel membrane-bound respiratory complex from *Desulfovibrio desulfuricans* ATCC 27774. *Biochim Biophys Acta - Bioenerg* 2003; **1605**: 67–82.
17. Watanabe T, Kojima H, Fukui M. Identity of major sulfur-cycle prokaryotes in freshwater lake ecosystems revealed by a comprehensive phylogenetic study of the dissimilatory adenylylsulfate reductase. *Sci Rep* 2016; **6**: 1–9.
18. Tan S, Liu J, Fang Y, Hedlund BP, Lian Z-H, Huang L-Y, et al. Insights into ecological role of a new deltaproteobacterial order *Candidatus* Acidulodesulfobacterales by metagenomics and metatranscriptomics. *ISME J* 2019; **13**: 2044–2057.
19. Haveman SA, Greene EA, Stilwell CP, Voordouw JK, Voordouw G. Physiological and Gene Expression Analysis of Inhibition of *Desulfovibrio vulgaris* Hildenborough by Nitrite. *J Bacteriol* 2004; **186**: 7944–7950.

20. Pereira IAC, Ramos AR, Grein F, Marques MC, da Silva SM, Venceslau SS. A comparative genomic analysis of energy metabolism in sulfate reducing bacteria and archaea. *Front Microbiol* 2011; **2**: 1–22.
21. Holkenbrink C, Barbas SO, Mellerup A, Otaki H, Frigaard N-U. Sulfur globule oxidation in green sulfur bacteria is dependent on the dissimilatory sulfite reductase system. *Microbiology* 2011; **157**: 1229–1239.
22. Dahl C, Engels S, Pott-Sperling AS, Schulte A, Sander J, Lübbe YJ, et al. Novel genes of the *dsr* gene cluster and evidence for close interaction of Dsr proteins during sulfur oxidation in the phototrophic sulfur bacterium *Allochromatium vinosum*. *J Bacteriol* 2005; **187**: 1392–1404.
23. Larsen Ø, Lien T, Birkeland N-K. Characterization of the desulforubidin operons from *Desulfobacter vibrioformis* and *Desulfobulbus rhabdoformis*. *FEMS Microbiol Lett* 2000; **186**: 41–46.
24. Debuscche L, Thibaut D, Cameron B, Crouzet J, Blanche F. Purification and characterization of cobyrinic acid *a,c*-diamide synthase from *Pseudomonas denitrificans*. *J Bacteriol* 1990; **172**: 6239–6244.
25. Fresquet V, Williams LK, Raushel FM. Mechanism of cobyrinic acid *a,c*-diamide synthetase from *Salmonella typhimurium* LT2. *Biochemistry* 2004; **43**: 10619–10627.
26. Raux E, Lanois A, Rambach A, Warren MJ, Thermes C. Cobalamin (vitamin B_{12}) biosynthesis: Functional characterization of the *Bacillus megaterium cbi* genes required to convert uroporphyrinogen III into cobyrinic acid *a,c*-diamide. *Biochem J* 1998; **335**: 167–173.
27. Zheng K, Ngo PD, Owens VL, Yang XP, Mansoorabadi SO. The biosynthetic pathway of coenzyme F_{430} in methanogenic and methanotrophic archaea. *Science* 2016; **354**: 339–342.
28. Buan NR, Metcalf WW. Methanogenesis by *Methanosarcina acetivorans* involves two structurally and functionally distinct classes of heterodisulfide reductase. *Mol Microbiol* 2010; **75**: 843–853.
29. Junier P, Junier T, Podell S, Sims DR, Detter JC, Lykidis A, et al. The genome of the Gram-positive metal- and sulfate-reducing bacterium *Desulfotomaculum reducens* strain MI-1. *Environ Microbiol* 2010; **12**: 2738–2754.
30. Romão C V, Archer M, Lobo SA, Louro RO, Pereira IAC, Saraiva LM, et al. Diversity of heme proteins in sulfate reducing bacteria. In: Kadish KM, Smith KM, Guilard R (eds). *Handbook of Porphyrin Science*, 19th ed. 2012. World Scientific Publishing Co. Pte. Ltd., Singapur, pp 139–230.
31. Sodergren EJ, DeMoss JA. *narI* region of the *Escherichia coli* nitrate reductase (*nar*) operon contains two genes. *J Bacteriol* 1988; **170**: 1721–1729.
32. Blasco F, Iobbi C, Ratouchniak J, Bonnefoy V, Chippaux M. Nitrate reductases of *Escherichia coli*: Sequence of the second nitrate reductase and comparison with that encoded by the *narGHJI* operon. *Mol Gen Genet* 1990; **222**: 104–111.
33. Löffler M, Wallerang KB, Venceslau SS, Pereira IACC, Dahl C. The Iron-Sulfur Flavoprotein DsrL as NAD(P)H:Acceptor Oxidoreductase in Oxidative and Reductive Dissimilatory Sulfur Metabolism. *Front Microbiol* 2020; **11**: 1–15.
